# Numerical and experimental study on turbulence statistics in a large fan-stirred combustion vessel

**DOI:** 10.1007/s00348-021-03212-9

**Published:** 2021-05-03

**Authors:** M. E. Morsy, J. Yang

**Affiliations:** 1 School of Mechanical Engineering, University of Leeds, Leeds, LS2 9JT United Kingdom; 2 Faculty of Engineering at El-Mattaria, University of Helwan, Cairo, 11718 Egypt

## Abstract

**Abstract:**

Particle image velocimetry (PIV) has become a popular non-intrusive tool for measuring various types of flows. However, when measuring three-dimensional flows with two-dimensional (2D) PIV, there are some uncertainties in the measured velocity field due to out-of-plane motion, which might alter turbulence statistics and distort the overall flow characteristics. In the present study, three different turbulence models are employed and compared. Mean and fluctuating fields obtained by three-dimensional computational fluid dynamics modeling are compared to experimental data. Turbulence statistics such as integral length scale, Taylor microscale, Kolmogorov scale, turbulence kinetic energy, dissipation rate, and velocity correlations are calculated at different experimental conditions (i.e., pressure, temperature, fan speed, etc.). A reasonably isotropic and homogeneous turbulence with large turbulence intensities is achieved in the central region extending to almost 45 mm radius. This radius decreases with increasing the initial pressure. The influence of the third dimension velocity component on the measured characteristics is negligible. This is a result of the axisymmetric features of the flow pattern in the current vessel. The results prove that the present vessel can be conveniently adopted for several turbulent combustion studies including mainly the determination of turbulent burning velocity for gaseous premixed flames in nearly homogeneous isotropic turbulence.

**Graphic abstract:**

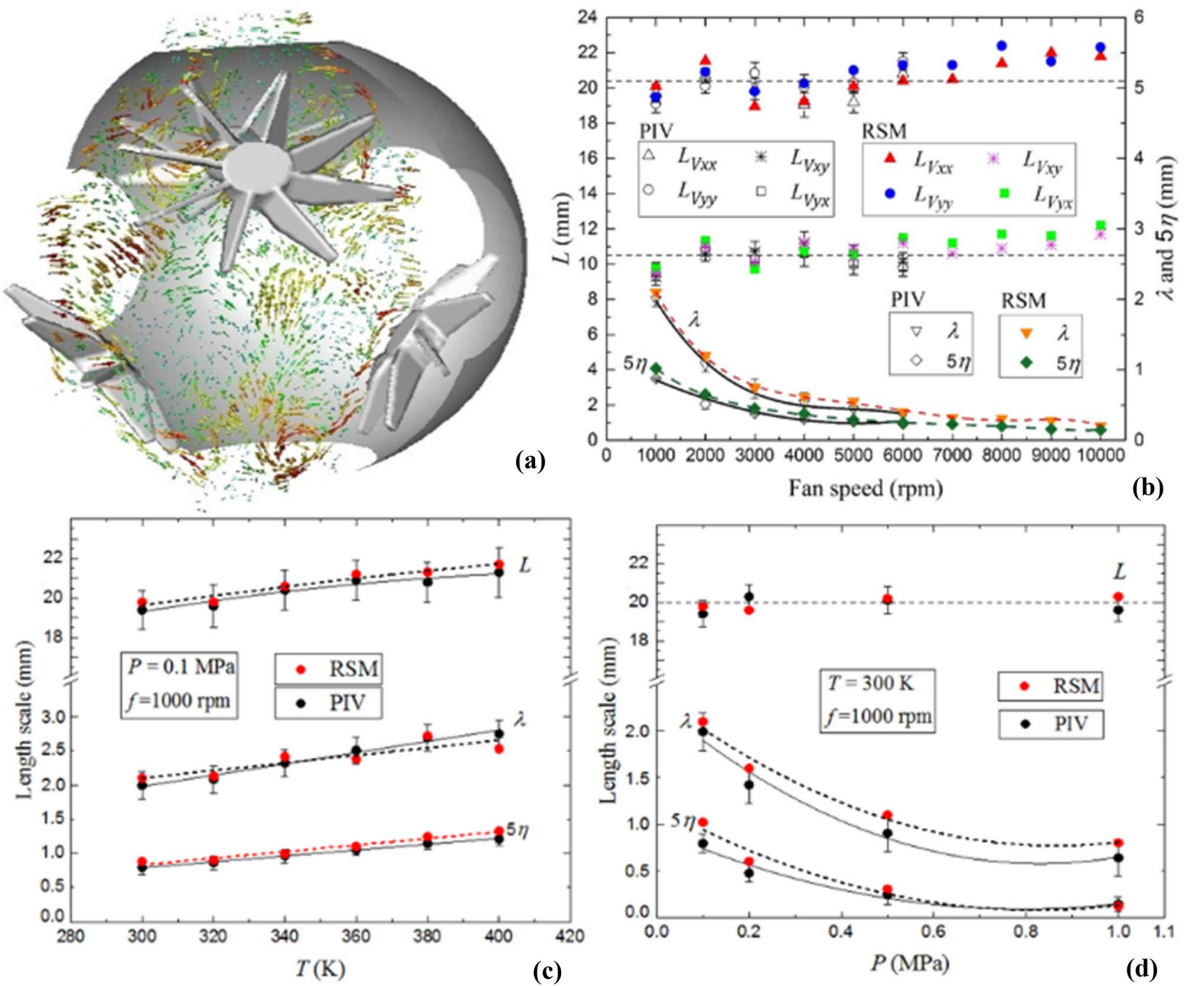

## Introduction

Turbulent burning velocity, ${u}_{t}$, is an essential parameter in the design and development of modern high turbulent combustors. An early critical reviews of ${u}_{t}$ showed that a spatially non-homogeneous mixture leads to changes in flame behavior in terms of heat release, propagation speed and consequently ${u}_{t}$ (Andrews et al., 1975; Semenov, 1965; Pasquier et al., 2007). Different effects induced by mixture inhomogeneities, such as variation in local reaction rate, interaction with turbulent flow, temperature and composition of burned gases, can lead individually to an increase or decrease in heat release and hence in ${u}_{t}$ (Jiménez et al., 2002). Bradley et al. (2003) have also shown that ${u}_{t}$ and flame wrinkling rate depend mainly on turbulence statistics, such as root-mean square of the turbulent velocity fluctuation, ${u}^{\prime},$ and turbulent length scale, *L*. Thus, accurate measurement of ${u}_{t}$ relies on the ability to obtain a nearly homogeneous and isotropic turbulence (HIT) with a well-defined turbulence statistics.

Different methods and experimental setups, pioneered by Schlossing and de Mondesir in 1864, see (Andrews et al. 1975), have been described in the literature in order to generate such a near-homogeneous isotropic turbulent flow. These methods include the use of fans (Semenov, 1965; Pasquier et al. 2007; Jiménez et al. 2002; Bradley et al. 2003; Ravi et al. 2013; O’neill et al. 2004; Sick et al. 2001; Weiß et al. 2008; Xu et al. 2017), loudspeakers (Hwang and Eaton, 2004; Goepfert et al. 2010), grid technology (Kang, et al. 2003) and multiple opposed jets (Xu et al. 2017). Vessels, within which turbulence is generated by several rotating fans, have been widely employed to generate HIT flow and to study the mutual interaction between flame propagation and turbulent flow (Krawczynski et al. 2006; Abdel-Gayed et al. 1984; Akindele et al. 1982; Dreizler et al. 2000; Fansler et al. 1990; Galmiche et al. 2014; Goulier et al. 2017a; Goulier et al. 2017b; Kwon et al. 1992; Lipatnikov et al. 2007; Wilcox, 1993). HIT conditions have been achieved in such vessels by controlling the turbulence parameters by multiple fans/impellers arranged in a regular tetrahedron or octahedron configuration. These configurations generate a central region of near-HIT conditions with negligible mean flow (Andrews et al. 1975; Birouk et al. 1996; Fallon et al. 2002).

Generating a high level of homogeneous isotropic turbulence inside fan-stirred vessels depends mainly on the size of the spherical cavity of the vessel and the fan geometric features. The vessel and windows must be large enough for a stable flame to be established and observed at near-constant pressure. This affects the initial characteristics of the turbulent flow, especially when dealing with turbulent premixed combustion. Several experimental studies have been conducted to prove the near-HIT flow fields in such vessels (Ravi et al. 2013; Weiß et al. 2008; Dreizler et al. 2000; Fansler et al. 1990; Galmiche et al. 2014, Goulier et al. 2017a; Goulier et al. 2017b). These studies have been limited by the size of the vessels which in turn affect the extent of the generated central homogeneous volume of isotropic turbulence inside the vessel. Due to these limitations, the generated turbulence may significantly suffer either from wall confinement effects or from very narrow turbulence spectrum. Such limitations are not existing in the current vessel which was designed aiming to achieve high homogeneity and isotropy in a large central region. Table 1 shows the historic development of fan-stirred vessels. This present one is probably the largest, with probably also the largest rms velocity, yielding a good indication of spatial distributions. Such a large size ensures minimal impact of wall confinement and radiation.

**Table 1:** Survey of some fan-stirred vessels, including present study

	Vessel geometry	Dimensions (mm)	Number of fans	Max. fan speed (rpm)	Max. $U^{\prime }$ (m/s)
Semenov, 1965	Spherical	$D$= 97	4	7000	10
Andrews et al. 1975	Cylindrical	$D$= 305, $l$ = 305	4	5000	4
Fansler et al. 1990	Cylindrical	$D=260$, $l= 260$	4	2500	2.2
Sick et al. 2001	Spherical	$D= 58$	4	7000	1.8
Weiß et al. 2008	Spherical	$D= 118$	4	10000	3.5
Ravi et al. 2013	Cylindrical	$D= 305$, $l= 356$	4	8300	1.7
Xu et al. 2017	Cubic	$l= 136$	2	2900	1.6
Present study	Spherical	$D= 380$	4	10000	12

Zhang et al. (2019) used the CFD to study the turbulent flow fields in a small stainless steel spherical vessel of 2.28 liters capacity and a diameter of 190 mm, with eight variable speed fans. Bonhomme et al (2014) computed the turbulent flow in a closed vessel stirred by six fans, with a diameter of 200 mm, at low levels of turbulence up to $u^{\prime }$=3 m/s. The present study provides a detailed characterization of the turbulence field inside a large fan-stirred spherical combustion vessel, with a diameter of 380 mm up to $u^{\prime }$= 10 m/s, in the absence of phase change and chemical reaction. CFD simulations and 2D PIV measurements have been employed to investigate the internal flow field, at different fan speeds. The goal is to improve the understanding of the flow structure and demonstrate the feasibility of CFD methods, under different operating conditions. The CFD allows also the investigation of the three dimensionality of the flow and its effect on the generated turbulence. The velocity fields obtained by the CFD are compared with the 2D PIV measurements.

Due to the rapid disappearance of the seeding particles, at high speeds, the PIV measurements were taken up to 6000 rpm. Experimental spatial and temporal fluctuations of the mean velocities and rms velocities are presented up to this speed. These results have been extended by the CFD modeling up to a fan speed of 10000 rpm, in which this limitation does not exist. The integral, Kolmogorov and Taylor length scales are evaluated from the calculated flow fields. Effects of pressure and temperature changes on such scales are presented at different fan speeds, using both PIV measurements and CFD modeling. Three different models, standard (STD) k-*ε* and Renormalization Group (RNG) k-*ε* and Reynolds Stress Equation Model (RSM), have been employed to investigate the flow behavior in the third direction, which is not possible using the 2D PIV. RSM model has been shown to be more reliable compared to other models. The results from the CFD modeling using RSM have been presented and compared with those of the PIV.

Because of the difficulties in measuring turbulence, and characterizing it, ahead of the spherical flames in explosions, the turbulence is often measured prior to explosion in the literature. In the present work, the PIV technique was employed to study the interaction between the flame and flow. Some preliminary PIV results from turbulent combustion experiments were presented. The results have shown that the radial flame propagation changes the original cold flow turbulence and there is a symbiotic relationship between the flow structures contained within the reactants and the propagating flame front. The flame is affecting the structure of the flow, and the flow is affecting the structure of the flame as shown in Sect. 5.

## Experimental apparatus

**Fig. 1:**
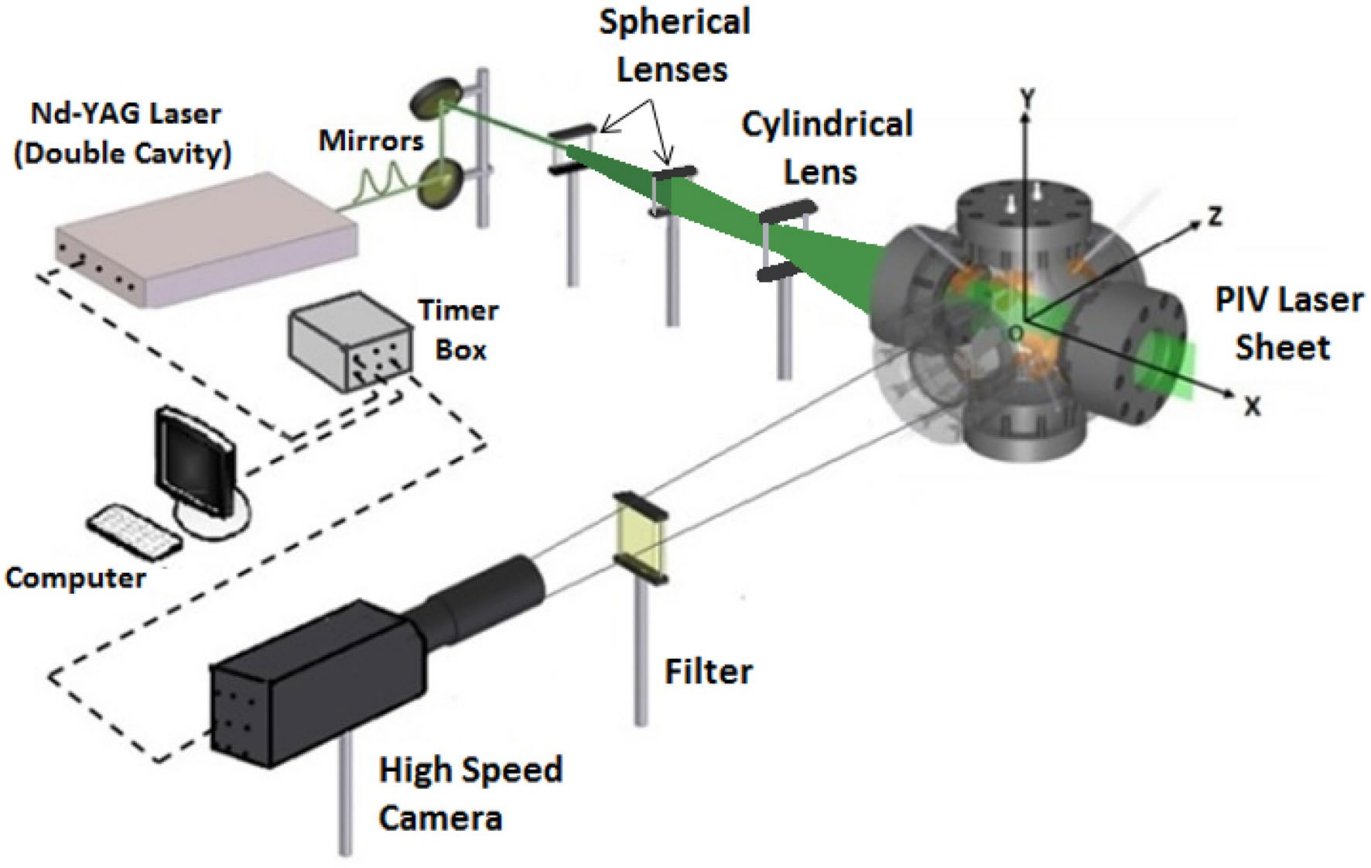
PIV optical setup and Leeds MK-II fan-stirred spherical combustion vessel

Measurements were taken in a spherical stainless steel explosion vessel with an inner diameter of 380 mm. The vessel is known as the Leeds MAKE 2 (MK-II) combustion vessel. The vessel has three pairs of optical quartzite windows of 150 mm diameter, allowing a full visualization of the center of the chamber, as shown in Fig. 1. Four fans, driven by 8 kW three-phase electric motors, are fitted and located close to the wall of the bomb in an orthogonal configuration, in order to produce a nearly homogeneous and turbulent flow. Each fan has 8 blades that are about 75 mm long and are about 72 mm apart at their edge. The blade pitch angle has not changed during the present work. All fans can be controlled individually with a set accuracy of 20–30 rpm.

Two pressure transducers were mounted flush with the inner surface of the vessel wall, one for static and the other for dynamic measurements in explosion studies. Only the static transducer was used, to study the effect of pressure on the turbulence characteristics. The vessel could be heated by an internal 2 kW coiled heating element, attached to the inside of the access cover. The temperature was set, controlled and displayed by a PID controller mounted in the control panel, using feedback via a K-type thermocouple (25 μm chromel-alumel wire). At a fan speed of 10000 rpm, the dissipation of energy increased the temperature of the air at 0.1 MPa by 3 K. A variable arc discharge ignition system with a centrally positioned spark plug, was used to ignite all reported mixtures in Sect. 5. The spark plug was designed and developed, at the University of Leeds, to minimize any aerodynamic interference with the flame. This spark was removed during the cold flow experiments. More details about the ignition system can be found in (Morsy, 2019).

A high repetition rate double-pulsed Nd:YAG laser (DM60-DH, Photonics), is employed to generate pulses of 12 mJ at a wavelength of 532 nm at 5 kHz. The laser beam is expanded into a vertical sheet of about 0.5 mm thickness, in passing through the center of the vessel, where it uniformly illuminates the dispersed seeding particles of olive oil, < 1 μm diameter, generated by six jet atomizers (9010F0021, DANTEC). The measuring system comprises a plano-concave and bi-convex spherical lenses of – 300, 650 mm focal lengths, respectively, with a plano-concave cylindrical lens of – 25 mm focal length. The laser pulses were synchronized with a high-speed camera perpendicular to the laser sheet, to record a 12-bit image pair of 1024 × 1024 pixels. The time between pulses was varied from 15 to 35 μs, depending on the fan speed. With this configuration, it was possible to measure the instantaneous velocities of the flow within a radius of 60 mm, from the vessel center. Each experiment was undertaken during about 2.5s, with the collection of an average of 12,500 images. An adaptive algorithm within the Dantec software, processed the images, with a minimum interrogation area of (16 × 16 pixels) and a maximum of (32 × 32 pixels), with a magnification ratio of 0.12 mm/pixel. The error in the case of out-of-plane motion is dominated by overlapping particles which change their intensity from bright–weak to weak–bright as they move together through the light sheet, as indicated by (Wieneke, 2015). This uncertainty should be minimal (less than 0.05 pixel) with (32 × 32 pixels) windows. Increasing the fan speed, increases the uncertainty of out-of-plane motion. An advanced processing algorism, namely adaptive PIV algorithm, has been employed to reduce such error. This adaptive PIV algorithm was an iterative and automatic way of calculating velocity vectors, based on the seeding particle density. The size and shape of individual interrogation areas, IA, were iteratively adjusted to fit the local seeding densities and velocity gradients. The appropriate IA size was automatically determined for each individual IA, by specifying maximum and minimum size limits. A first iteration always used the largest IA size, which was reduced in subsequent iterations. This allowed reduction of IA sizes where the particle density was sufficiently high. The minimum IA determined the location and magnitude of vectors, and how close to the borders a vector might be located. Further details about the PIV system, its optical configuration and the adaptive algorithm are to be found in (Bradley et al. 2019).

## Numerical methodology

To predict the turbulent flow field inside MK-II vessel, it is necessary to solve the mass conservation and momentum conservation formulae (so-called Navier–Stokes equations) for the fluid domain. Ideally Direct Numerical Simulation (DNS) should be applied, i.e., the Navier–Stokes equations should be solved without recourse to turbulence modeling, to resolve the fluid motion across the full ranges of spatial and temporal scales. In order to achieve this, the computational grid must be sufficiently fine to resolve the flow down to the Kolmogorov scale, which depends on the average rate of energy dissipation per unit mass and kinematic viscosity. However, the computational cost of DNS is prohibitively expensive for the application of fan-stirred MK-II vessel (where the Reynolds number, *Re*, of flows generally exceeds O(10)^4^ , under atmospheric conditions.

Another approach involves the numerical solution of the Reynolds-averaged (or time-averaged) N-S (RANS) equations together with an additional turbulence model, e.g., eddy-viscosity or Reynolds stress models. Traditionally, a turbulent flow is viewed as a field of fluctuations embedded in a mean flow. Accordingly, all variables pertaining to the flow (e.g., velocity, pressure, etc.) can be split into a mean and a deviation from the mean. A mean value for each variable can then be obtained by means of time averaging. The present predictions were based on the solutions of the incompressible Reynolds-averaged Navier–Stokes (RANS) equations using the following expressions:

1 $$\frac{\partial\uprho }{\partial {\rm t}}+\sum_{{\rm i}=1}^{3}\frac{\partial }{\partial {{\rm x}}_{{\rm i}}}\left(\uprho {\stackrel{-}{{\rm u}}}_{{\rm i}}\right)=0$$

2 $$\begin{aligned} \frac{\partial }{{\partial t}}\left( {\bar{u}_{i} } \right) & + \sum\limits_{{j = 1}}^{3} {\frac{\partial }{{\partial x_{j} }}} \left( {\rho \bar{u}_{i} \bar{u}_{j} } \right) \\ & = - \sum\limits_{{i = 1}}^{3} {\frac{{\partial \bar{p}}}{{\partial x_{i} }}} + \sum\limits_{{j = 1}}^{3} {\frac{\partial }{{\partial x_{j} }}} \left[ {\left( {\frac{{\partial \bar{u}_{j} }}{{\partial x_{i} }} + \frac{{\partial \bar{u}_{i} }}{{\partial x_{j} }} - \frac{2}{3}_{{ij}} \sum\limits_{{k = 1}}^{3} {\frac{{\partial \bar{u}_{k} }}{{\partial x_{k} }}} } \right)} \right] \\ & + \sum\limits_{{j = 1}}^{3} {\frac{\partial }{{\partial x_{j} }}} (\rho u^{\prime } _{i} u^{\prime } _{j} ) \\ \end{aligned}$$

where $\rho , \mu ,P$ and $u$ are the gas density, viscosity, pressure and velocity, respectively. Symbols overbar ( ¯ ) and prime ( ' ) indicate the mean and the fluctuating component. Subscripts *i*, *j* and *k* are the vector components in the *i*^th^, *j*^th^ and *k*^th^ directions, respectively. The unknown term $(-\rho \stackrel{-}{{u}_{i}^{{\prime}}{u}_{j}^{{\prime}}})$ in Eq. (2) is so-called Reynolds stress tensor and will generally be nonzero. Solving for this unknown is non-trivial and is the key problem in turbulent flow modeling. The eddy viscosity models assume the turbulent stress is proportional to velocity gradient with proportional constant µ_t_. Once the turbulent viscosity µ_t_ is know, τ_ij_ can be calculated. A notable example is the k-*ε* model (Launder and Spalding 1972) in which µ_t_ is correlated to the turbulent kinetic energy, k, and the turbulent dissipation rate, *ε*. Solving these two transport equations (i.e., k-equation and *ε*-equation) makes it possible to compute µ_t_, which in turn makes it possible to obtain the Reynolds stress tensor. The standard k-ε model was initially proposed for modeling steady flows with high Reynolds numbers and was considered unsuitable for “low Reynolds number" zones and transient flows. The model cannot predict anisotropies of normal Reynolds stresses. To overcome this deficiency, the RNG k-ε model was proposed. This model incorporates a new rate-of-strain term into the ε-transport equation. The interested reader is referred to (Orszag, 1993) for details.

In contract, Reynolds Stress Model (RSM) (Launder et al. 1975) closes RANS equations by solving transport equations (5 PDEs for 2D flow or 7 PDEs for 3D flow) for the Reynolds Stress terms instead of eddy-viscosity hypothesis. These transport equations consist of pressure-strain term, dissipation term, convection term, diffusion term and production term, which account for the effect the change of strain rare, system rotation and curvature. Therefore, RSM gives accurate predictions for complex flows, e.g., swirling, rotation, acceleration and retardation. In addition, the standard wall function was employed to resolve the viscosity-affected near-wall region. In which Reynolds stress components and turbulence dissipation rate were derived from the turbulence intensity and characteristic length via algebraic equations. The k-equation was used to compute the kinetic energy in this region. In the bulk flow, transport equations for Reynolds stresses and turbulence dissipation rate were solved, and the turbulence kinetic energy takes the trace of the Reynolds stress tensor. A comprehensive description on all transport equations of RSM model and wall function would be beyond the scope of this work. The interested reader is referred to (ANSYS, 2020) for details. Though RSM solves Reynolds stresses using transport equations, the instantaneous velocity fluctuation (presented in Sect. 4) was derived from temporally developing flow field as unsteady RANS methods. However, RSM would be rather laborious and time-consuming since there are more transport equations to implement and solve. Overall, RANS-based modeling allows the use of rather coarse grids, and thus greatly reduces the required computational costs. Therefore, it is the most widely used in academic research. For example, Elbadawy et al. (2015) investigate the spray characteristics in a spherical vessel, using k-ε model. However, the swirling flow induced by the stirred-fan was simplified as inlet flow motions.

A further option is to use a Large Eddy Simulation (LES) approach to resolve the largest structures in the flow field, and account for sub-grid scale eddies using appropriate models. LES is considered as an attractive method since it provides a satisfactory compromise predictive accuracy and computational costs. Although a couple of successful examples of its use in studies on small fan-stirred combustion vessel have been reported in the literature (Zhang, 2019; Bonhomme et al., 2014), LES modeling on MK-II which features the largest volume (inner diameter 380 mm) still challenges the capabilities of existing computational resources.

**Fig. 2:**
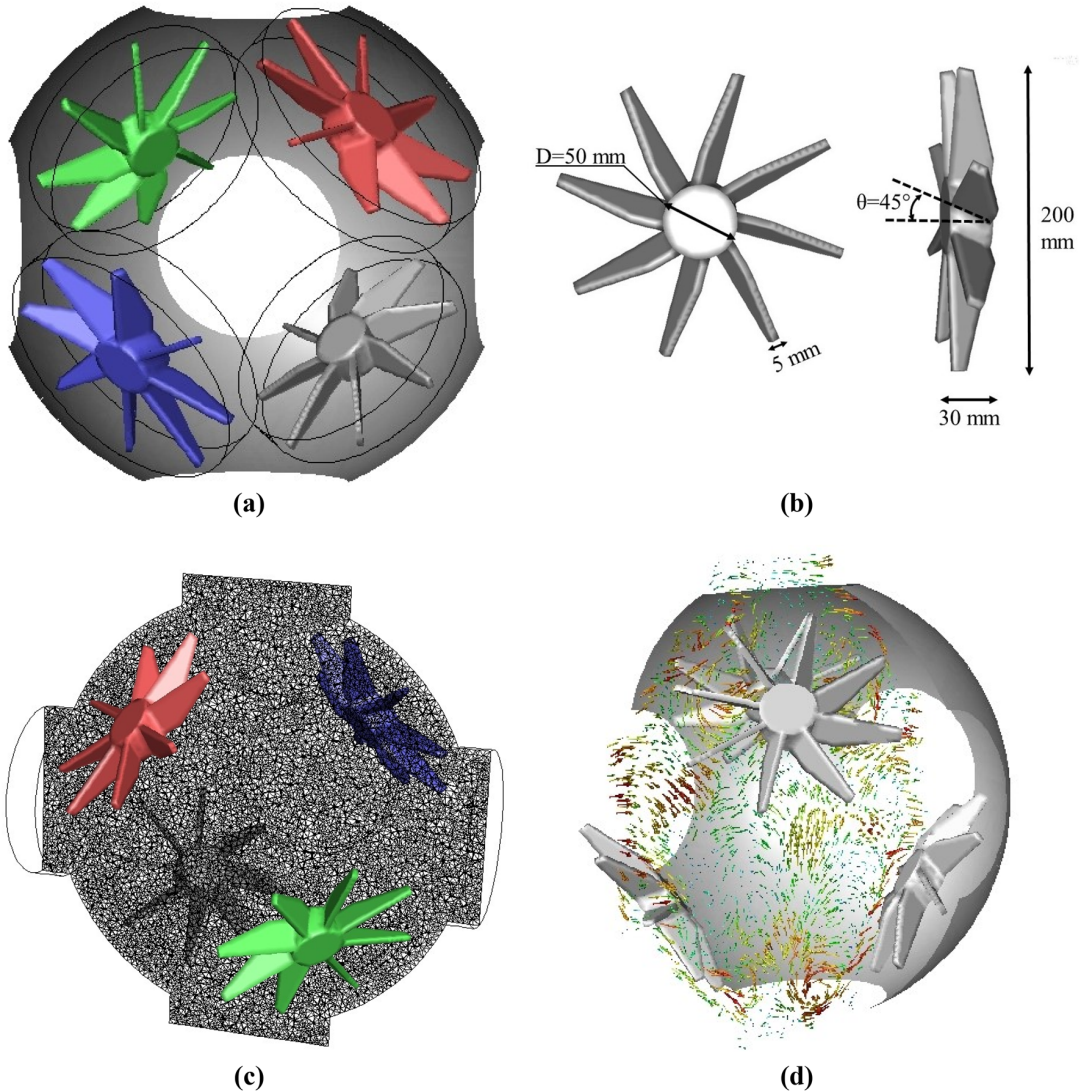
**a** Inner domain of MK-II, **b** Dimensions of stirred fan, **c** Geometry of internal grids along a section plane that passes through the middle of domain, **d** Velocity vector field on the cross-sectional plane obtained from Reynolds Stress modeling

In the CFD-based approach used in the simulations reported here, all the above governing equations together with transport equations were discretized and solved simultaneously using the commercial code ANSYS Fluent ver. 19.0 (ANSYS, 2020). A 3D computational domain, consisting of ~8 million unstructured hexahedral/tetrahedral cells, was constructed using the commercial mesh generator ICEM CFD. The grid size varies from 2 mm (the core region and near fan region) to 5 mm (the rest of domain). The whole domain consisted of four cylindrical rotating sub-domains for fans and one stationary sub-domain for the rest of MK-II vessel. Each cylindrical domain features a diameter of 210 mm and height of 35 mm which fits individual fan (see Fig. 2a). The characteristics of stirring fan were provided in Fig. 2b. The fluid zone adjacent to the fan surface in this sub-domain was assigned to move at the same speed as the fan. The transient fluid interaction between rotating part and stationary part was achieved via sliding mesh technique. A section plane that passes through the middle of domain was created to show the geometry of internal grids, see Fig. 2c. An example of velocity vector on this cross-sectional plane was displayed in Fig. 2d. A no-slip condition was imposed on both moving and stationary walls. The rotation speed of fan was varied from 1000 to 10000 rpm and the corresponding rotation period was 5s. The initial conditions were selected to represent practical conditions in MK-II, e.g., a range of temperatures (300–400 K) and pressures (0.1–1 MPa) were considered.

The pressure-velocity coupling was handled by the Semi-implicit Method for Pressure-linked Equation (SIMPLE) algorithm, while second order and bounded central differencing schemes were used as the discretization methods for pressure and momentum, respectively. The second order implicit method was used for temporal discretization. To ensure accuracy, the Courant number was kept to less than 0.25 for the core flow region (diameter 120 mm) by choosing a fixed time step of 0.001s and a maximum number of 20 iterations for each time step. The turnaround time for the simulations of 5 seconds flow time was ~74 h on a Linux based High Performance Computing cluster run in parallel over 8 processors.

## Results and discussion

### Mean and rms velocities

**Fig. 3:**
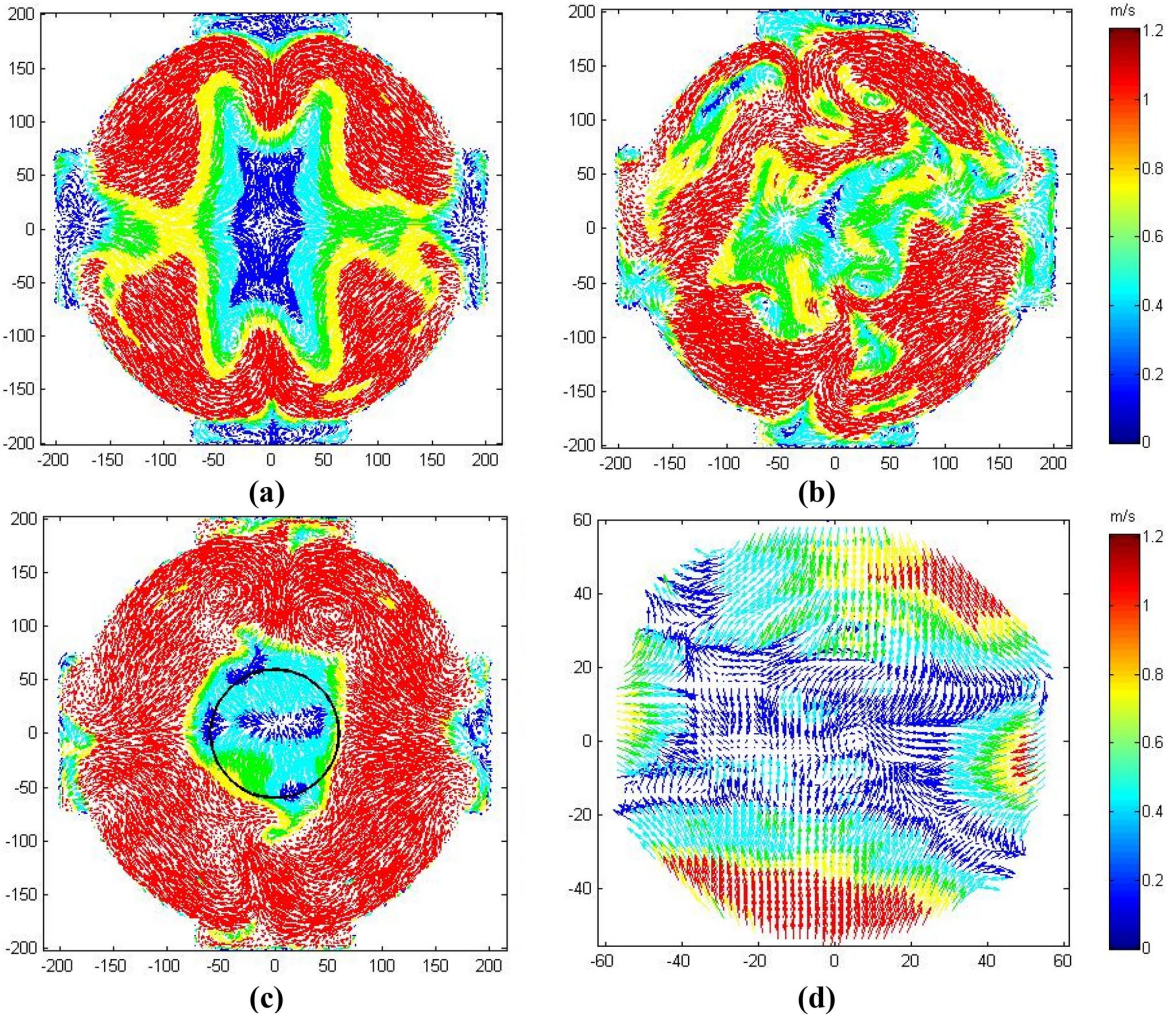
Time-averaged local mean vector fields at the central cross section of MK-II for fan speed 5000 rpm; obtained by **a** STD (k-*ε*) model, **b** RNG (k-*ε*) model, **c** RSM modeling and **d** PIV measurement, at T = 300 K and P = 0.1 MPa

Figure 3 shows time-averaged local mean vector fields at fan speed, 5000 rpm, for $T$ = 300 K and P = 0.1 MPa, using STD (k-*ε*) , RNG (k-*ε*), RSM modeling and PIV measurements. For clarity, only half the vectors are displayed, with the same color bar to indicate the difference between velocity maps. All measurements geometrically employed the same plane, passing through the center of the vessel. All maps show an increase of the velocity from the center of the chamber toward the wall. However, there is a similarity in flow patterns, and the mean velocities are close to zero in the central region and slightly increases toward the wall but remain below 1.0  m/s up to a radius of ~ 50 mm.

This value is less than 10% of the mean rms velocity, at the same fan speed, and shows that the isotropy and homogeneity are particularly good in the central region.

Figure 3 shows the comparison of predicted and measured mean velocity field at the central cross section of MK-II. The time periods for temporal averaging were 5 seconds and 2.5 seconds for RANS-based models (Fig. 3a-c) and PIV technique (Fig. 3d). Within the view filed of interest indicated by the centralized circled region (±60 mm), a close agreement between the PIV mean velocities and that of the RSM model was observed in terms of velocity magnitude and flow structure. Note that RANS-based models calculate the ensemble-averaged flow field. It would be more appropriate to employ DNS or LES to solve instantaneous flow fields if sufficient computing resources were available.

**Fig. 4:**
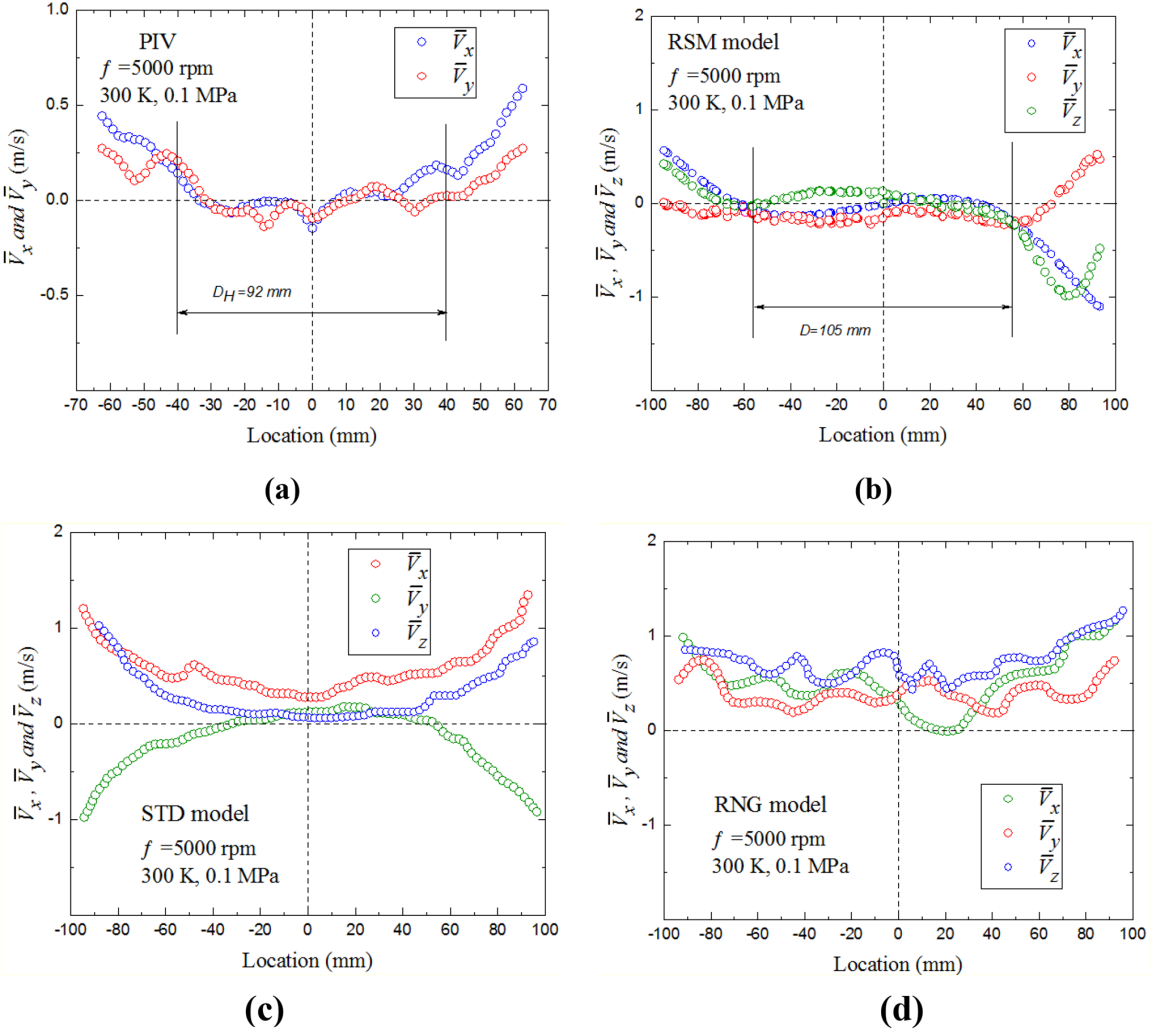
Spatial variations of mean velocity components along x-, y- and z-axes, at fan speed of 5000 rpm, **a** PIV, **b** RSM model, **c** STD model and **d** RNG model

A quantitative comparison of velocity magnitudes within that circle obtained by PIV and RSM is shown in Figs. 4a and 4b, under the same conditions of Fig. 3. The velocity component ${\bar{V }}_{x}$ was calculated along the x-axis for y = 0, while ${\bar{V }}_{y}$ was calculated along the y-axis for x = 0 in the x-y plane, passing through the central region of the vessel. The velocity component ${\bar{V }}_{z}$ was calculated along the z-axis for y = 0 in the z-y plane, normal to the PIV plane. In Fig. 4a, the variations of the PIV two velocity components are small and their fluctuations are almost uniform, within a distance of ±60 mm, indicative of a high level of isotropy. Slight increases in velocities, of less than 0.6 m/s, are observed beyond a radius of  ± 46 mm. Nevertheless, the values of the two components are small when compared with the rms velocity. In Fig. 4b, the variations of the RSM velocity components, ${\bar{V }}_{x}$,${\bar{V }}_{y}$ and ${\bar{V }}_{z}$, are also small and close to zero, within a radius of ±52.5 mm. The velocity component, ${\bar{V }}_{z}$, is consistent with the other velocity components ${\bar{V }}_{x}$ and ${\bar{V }}_{y}$, indicating a symmetrical flow structure in the three dimensions, due to using four identical fans arranged in a regular tetrahedron configuration in a spherical vessel. Figures 3 and 4 show that the flow structure and velocity magnitudes are similar to each other. This provides verification that the bomb model is well constructed, and that the PIV is working properly. Figure 4c and d show similar comparison between the PIV and other two models (i.e., STD (k-*ε*) and RNG (k-*ε*)). Such comparison has shown less agreement, with high mean velocities at the central volume of the bomb.

**Fig. 5:**
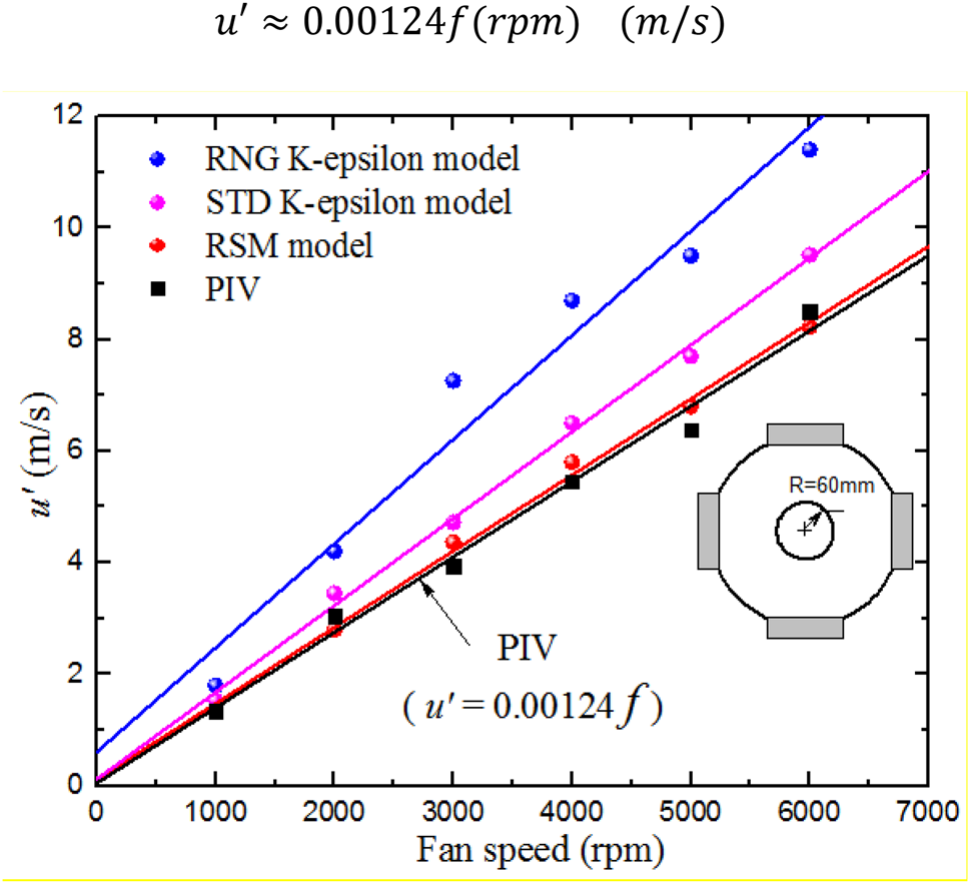
Effect of fan speed on the turbulence rms velocity, ${u}^{{\prime}}$. The predicted and measured data points (discrete symbols) are accompanied by the best fit curves

The values of ${u}^{\prime}$ were estimated for each interrogation area and then averaged over all the interrogation areas within the area of interest, using PIV, STD (k-*ε*), RNG (k-*ε*), RSM. These values are plotted in Fig. 5. Close agreement is observed between PIV and RSM, with larger differences between experiments and other CFD models. Despite the small differences in ${u}^{\prime}$ between the RSM and measured data, the proposed numerical simulation, which consider the full scale of the vessel and dynamic motion of four rotating fans, is able to reproduce similar behaviors for the turbulence intensity as observed in experiments. A linear fit applied to the RSM and PIV measurements yields a correlation between rms turbulent velocity and fan speed, see Eq. (3). In addition, the results prove that the RSM model is superior to the other two models when dealing with the rotating flows in the combustion vessel. Therefore, the results reported in the following sections focused on PIV and RSM.

3 $$u^{\prime} \approx {\text{0}}.{\text{00124f}}({\text{rpm}})({\text{m/s}})$$

### Diameter of homogeneity

**Fig. 6:**
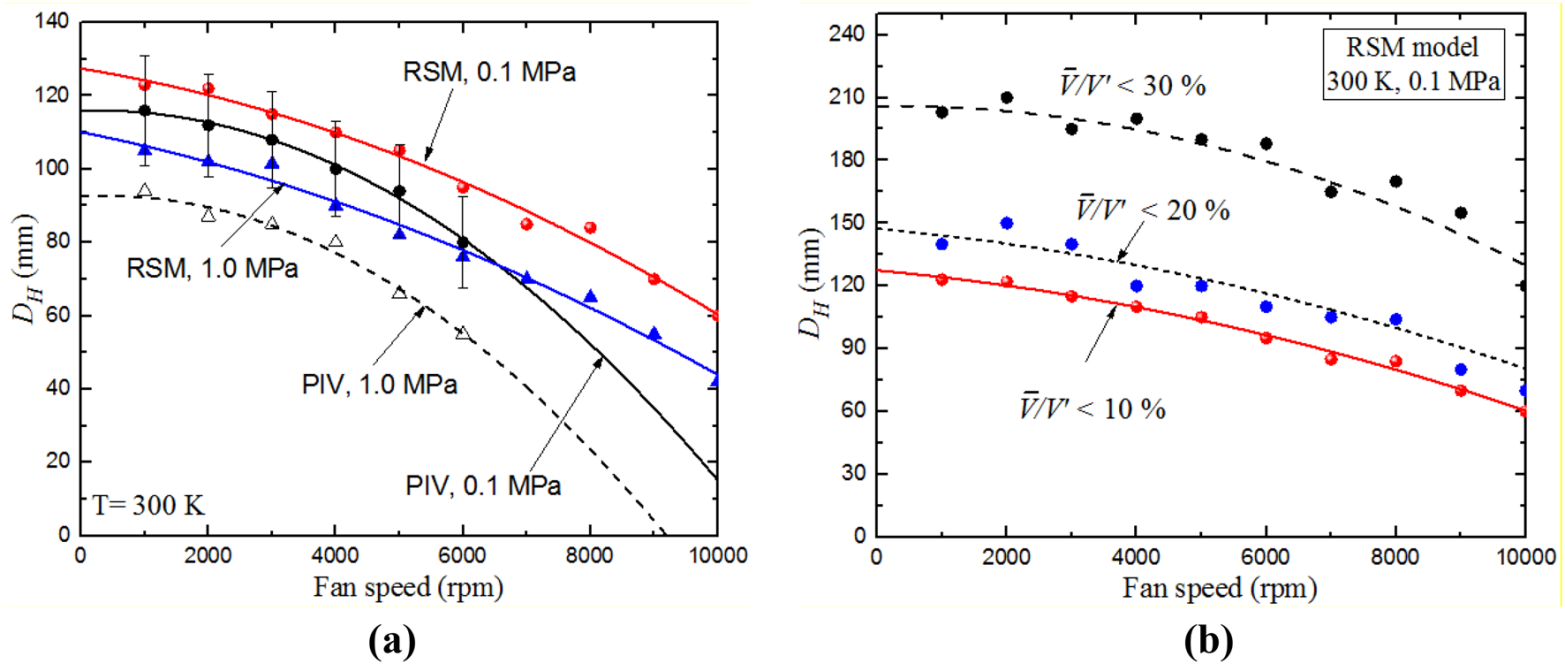
Effect of fan speed on the radial extent of homogeneous, isotropic turbulence **a** at different pressures **b** at different percentages of $\bar{V }/V{^{\prime}}$ . The predicted and measured data points (discrete symbols) are accompanied by the best fit curves. Arrows indicate the condition of each curve

Similar to previous studies of (Semenov, 1965; Ravi et al. 2013; Sick et al. 2001), the homogeneous area can be characterized by (${\bar{V }}_{x}<10\%{V}_{x}^{{\prime}}$ and ${\bar{V }}_{y}<10\%{V}_{y}^{{\prime}}$). Mean and rms velocities have been calculated at each IA, and the corresponding area which satisfy these conditions has been assigned. Figure 6a shows the radial diameter,${D}_{H}$, within which the flow is homogeneous and isotropic, for different fan speeds at 0.1 and 1.0 MPa and 300 K. Both PIV and RSM show that ${D}_{H}$ decreases with fan speed and pressure, resulting in smaller area of homogeneity and isotropy. Due to the limited area of PIV measurements, $\bar{V}/V^{\prime}$ was calculated using the RSM results for the full scale of the vessel. This value is still low (< 30%) for the whole area, as shown in Fig. 6b.

### PDFs of turbulent velocities

A homogeneous turbulence was characterized by a turbulent flow field exhibiting a reasonably Gaussian PDF of velocity fluctuations (Krogstad and Davidson, 2010, 2011). It was also described by the same PDF throughout the flow and that exhibits invariance with the variation of spatial position (Batchelor, 1953). Accordingly, turbulent velocity fluctuations ${V}_{xN}$ and ${V}_{yN}$ about the mean, were normalized by the local rms value and were calculated as in (Galmiche et al. 2014). In x-direction, ${V}_{xN}$ is given by:

4 $${V}_{xN}\left(x,y,i\right)=[{V}_{x}\left(x,y,i\right)-\stackrel{-}{{V}_{x}}\left(x,y\right)]/{{V}_{x}}^{{\prime}}\left(x,y\right).$$

**Fig. 7:**
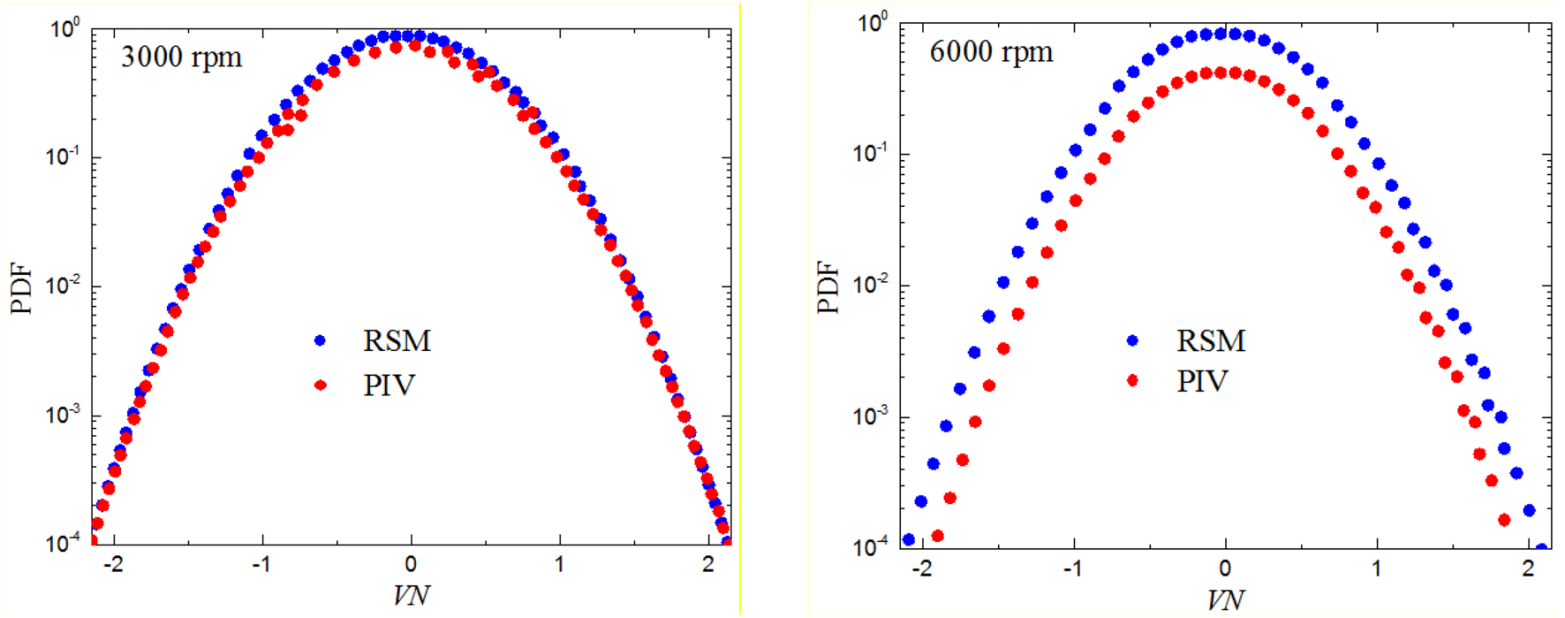
Comparison of predicted (red circles) and measured (blue circles) PDFs of normalized fluctuating velocities ${V}_{{\rm N}}$ at fan speeds 3000 rpm (left) and 6000 rpm (right) with a pressure 0.1 MPa and temperature 300 K

${V}_{yN}$ can be calculated using Eq. (4), by replacing ${V}_{x}$, $\stackrel{-}{{V}_{x}}$ and ${{V}_{x}}^{{\prime}}$ by ${V}_{y}$, $\stackrel{-}{{V}_{y}}$ and ${{V}_{y}}^{{\prime}}$, respectively. The PDFs of normalized mean velocity, V_N_, of ${V}_{xN}$ and ${V}_{yN}$ , for fan speeds of 3000 and 6000 rpm are shown in Fig. 7 , along with the theoretical Gaussian distribution, demonstrating satisfactory agreements at 3000 rpm with the PDFs are slightly peaked at 6000 rpm. The PIV and RSM results appear to be consistent with slight difference at the high speed.

The Gaussian distribution of all PDFs was further checked by evaluating the skewness factor, ${S}_{k}$, and the kurtosis factor, $K$. The Skewness factor is the third moment of ${{V}_{x}}^{\prime}$ or ${{V}_{y}}^{\prime}$ normalized by the variance. With a symmetric distribution, the skewness factor, ${S}_{k}$, is equal to zero. For ${S}_{k}$ 0, positive fluctuations are dominant. For ${S}_{k}<0$ negative fluctuations dominate. ${S}_{k}$ measures the asymmetry of the distribution. The Kurtosis factor,$K,$, is the 4th moment of ${{V}_{x}}^{\prime}$ or ${{V}_{y}}^{\prime}$ normalized by the variance and is another descriptor of the shape of a probability distribution. For a Gaussian distribution, $K$ is equal to 3. A distribution characterized by lower or higher $K$ implies a shorter or longer tail, respectively, than the Gaussian one. In $x$-direction, ${S}_{k}$ and $K$ are given by (Galmiche et al. 2014):

5 $${S}_{k,{V}_{x}}= \frac{1}{n}\sum_{j=1}^{n}\left(\frac{\frac{1}{{N}_{im}}\sqrt{\sum_{i=1}^{{N}_{im}}{\left[{V}_{x}\left(x,y,i\right)-\stackrel{-}{{V}_{x}}\left(x,y\right)\right]}^{3}}}{{[{{V}_{x}}^{{\prime}}\left(x,y\right)]}^{3}}\right).$$

and

6 $${K}_{{V}_{x}}= \frac{1}{n}\sum_{j=1}^{n}\left(\frac{\frac{1}{{N}_{im}}\sqrt{\sum_{i=1}^{{N}_{im}}{\left[{V}_{x}\left(x,y,i\right)-\stackrel{-}{{V}_{x}}\left(x,y\right)\right]}^{4}}}{{[{{V}_{x}}^{{\prime}}\left(x,y\right)]}^{4}}\right).$$

Here $n$ is the total number of grid nodes in the vector map. The corresponding skewness and kurtosis in the $y$-direction, respectively, noted ${S}_{k,{V}_{y}}$ and ${K}_{{V}_{y}}$, are calculated in the same way, by using ${V}_{y}$, $\stackrel{-}{{V}_{y}}$ and ${V}_{y}^{\prime}$, in Eqs. (5) and (6), instead of ${V}_{x}$, $\stackrel{-}{{V}_{x}}$ and ${V}_{x}^{\prime}$, respectively.

**Fig. 8:**
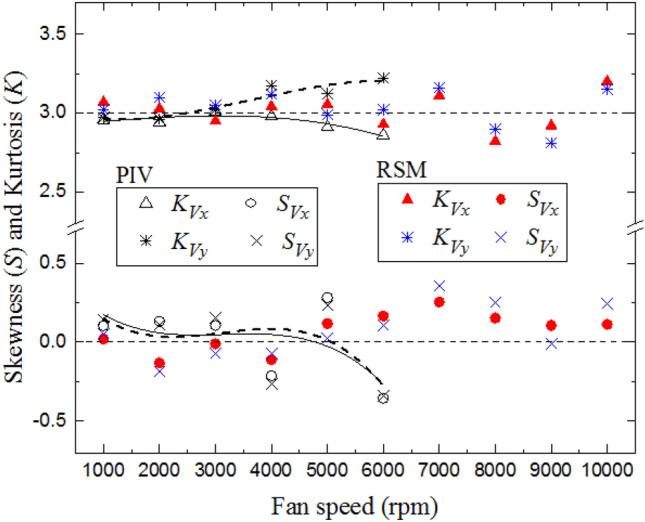
Comparison of predicted and measured skewness, *S*_*v*_, and kurtosis factors, *K*_*v*_, as a function fan speeds at 0.1 MPa and 300 K. The discrete data points are accompanied by the best fit curves

Figure 8 shows the variations in the skewness factors, ${S}_{{V}_{x}}, {S}_{{V}_{y}}, {K}_{{V}_{x}}$ and ${K}_{{V}_{y}}$ as a function of the fan speed. The solid and dotted curves are the best fit curve to the experimental PIV results. Due to the rapid disappearance of the seeding particles at high speed, the PIV measurements were limited by a fan speed of 6000 rpm. The results of the PIV measurements were extended by that of RSM to a fan speed of 10000 rpm. Both PIV and RSM results show that the skewness values are close to zero and the kurtosis is reasonably close to 3.0, which highlights the independency of these statistical factors to the variation with the fan speed. The results confirm these features of homogeneous and isotropic flow, within the defined regions.

### Characteristic length scales

Based on the calculated resolved flow velocities from RSM and measured data from PIV, the spatial longitudinal and lateral integral length scales were evaluated directly from the integral of the correlation coefficients, ${R}_{{V}_{{\rm xx}}}$, ${R}_{{V}_{{\rm yy}}}$ and ${R}_{{V}_{{\rm yy}}}$, ${R}_{{V}_{{\rm yx}}}$, respectively. In *x*-direction, ${R}_{{V}_{{\rm xx}}}$ and ${R}_{{V}_{{\rm yx}}}$ are calculated as (Galmiche et al. 2014):

7 $${R}_{{V}_{{\rm xx}}}\left(\xi \right)=\frac{\langle {V}_{x}\left(x,y\right){V}_{x}(x+\xi ,y)\rangle }{{{{V}_{x}}^{{\prime}}}^{2}}, { L}_{{V}_{{\rm xx}}}={\int }_{0}^{{R}_{0}}{R}_{{V}_{{\rm xx}}}\left(\xi \right) {\rm d}\xi .$$

8 $${R}_{{V}_{{\rm yx}}}\left(\xi \right)=\frac{\langle {V}_{y}\left(x,y\right){V}_{y}(x+\xi ,y)\rangle }{{{{V}_{y}}^{{\prime}}}^{2}}, { L}_{{V}_{{\rm yx}}}={\int }_{0}^{{R}_{0}}{R}_{{V}_{{\rm yx}}}\left(\xi \right) {\rm d}\xi .$$

**Fig. 9:**
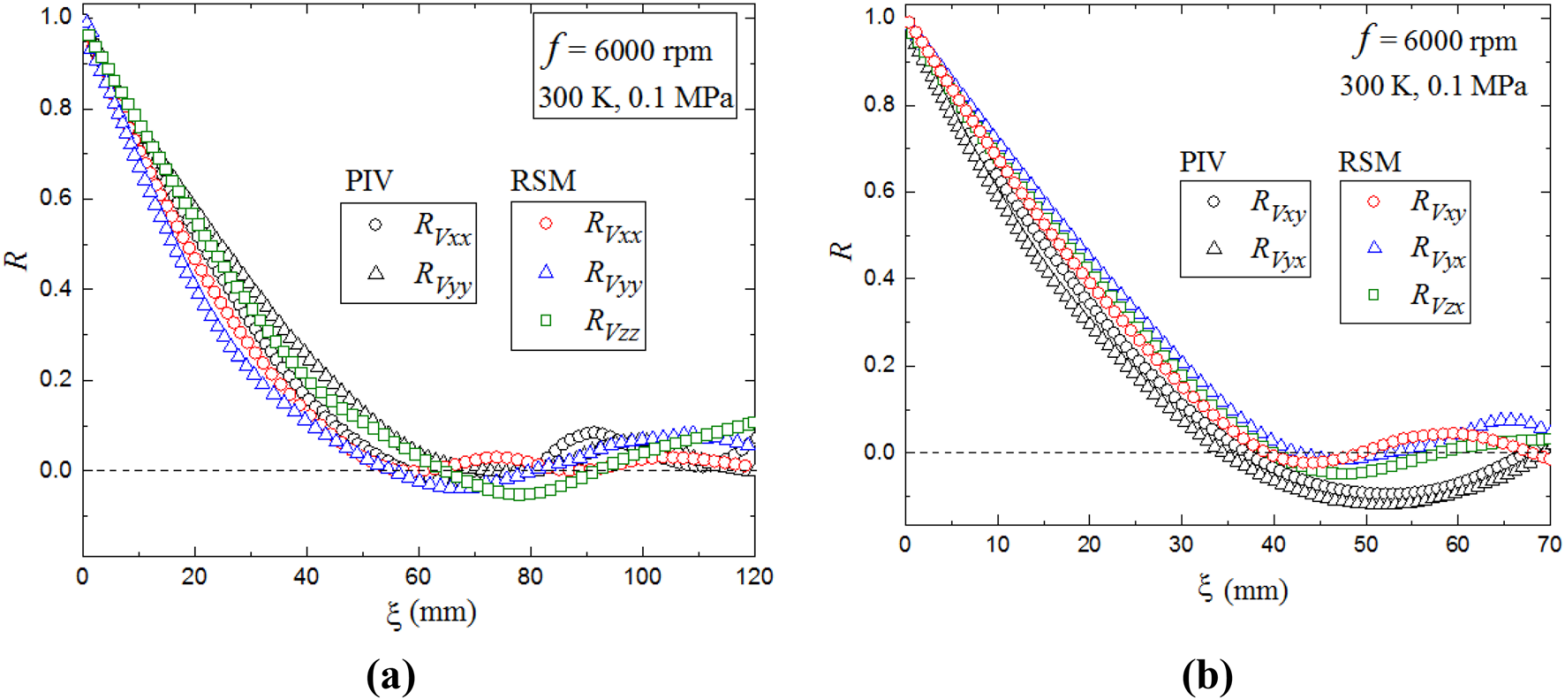
Comparison of predicted and measured spatial correlation coefficients, *R*_*v*_: **a** longitudinal component, **b** transverse component at the condition of 6000 rpm, 0.1 MPa and 300 K

Equations (7) and (8) are employed to calculate ${R}_{{V}_{xy}}$ and ${R}_{{V}_{yy}}$, in $y$-direction (Galmiche et al. 2014). Figure 9a shows that the autocorrelation functions decrease as the spatial distance, $\xi$, increases. The integral length scale, which captures the largest eddies containing most of the turbulent kinetic energy, is typically computed based on the integration of the autocorrelation function, $R$, up to the point where it reaches its minimum value (Ravi et al. 2013; Hwang and Eaton. 2004). The differences between the two longitudinal correlation coefficients ${R}_{Vxx}$ and ${R}_{Vyy}$ are small and similar to the differences between the lateral coefficients ${R}_{Vxy}$ and ${R}_{Vyx}$, shown in Fig. 9b, proving the near-isotropic nature of the flow field within the measurement area. The PIV and RSM results appear to be consistent again, confirming the ability of RSM to reproduce the experimental measurements with high accuracy. The correlation coefficient in z-direction, ${R}_{Vzz}$, is also constant with that of ${R}_{Vxx}$ and ${R}_{Vyy}$, confirming the negligible effect of the third dimension on the PIV measurements.

**Fig. 10:**
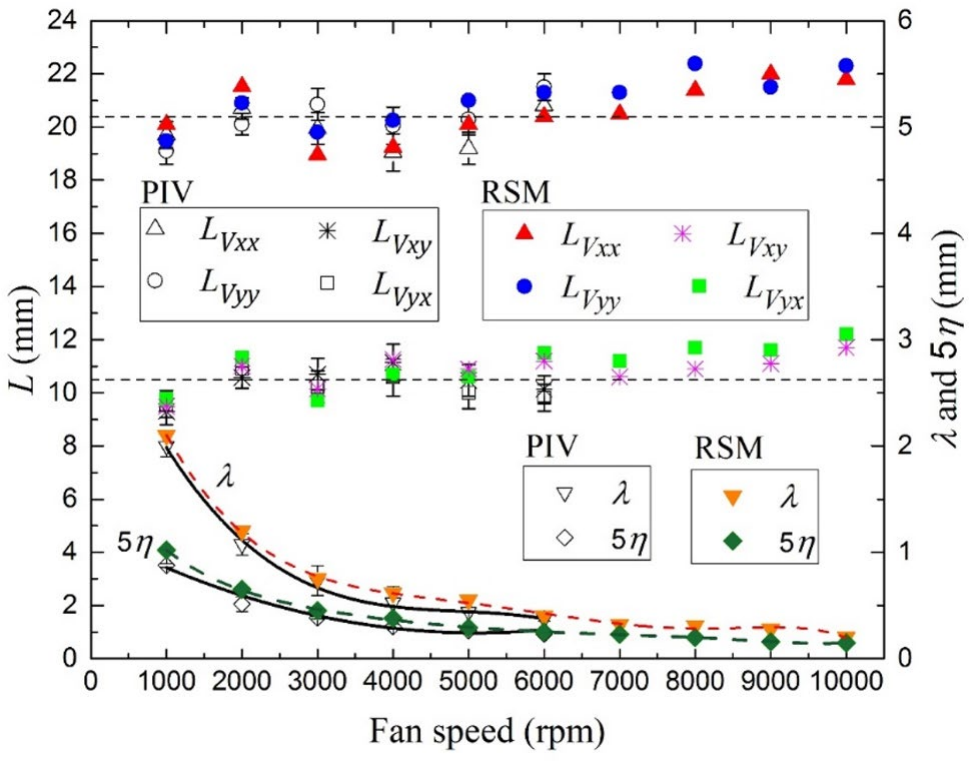
Comparison of predicted and measured integral turbulent length scales, *L*_*v*_ as a function of fan speeds at 0.1 MPa and 300 K. The derived Taylor length scales, λ, and five times Kolmogorov length scale, 5η, are shown at the bottom of graph

In Fig. 10, the longitudinal and lateral integral length scales show nearly constant values of ~ 20 and 10 mm, with slight increase at fan speed higher than 6000 rpm, and a good agreement between the results of the PIV and RSM. Error bars correspond to the standard deviation of the integral length scales determined from the two-point PIV velocity correlation curves of each instantaneous velocity map. These results show that integral length scale is independent of fan speed. Similar results are also commonly observed in (Ravi et al. 2013; Sick et al. 2001; Fansler et al. 1990; Galmiche et al. 2014) and in complete agreement with the conclusions of Fig. 10. In addition, the error bars of the PIV measurement due to the out-of-plane motion were provided for all mean values.

Due to the finite resolution of the PIV and RSM modeling, the Taylor length scale, $\lambda$, is calculated, based on a correction method to the dissipation rate,$\varepsilon$, which has been proposed by (Lavoie et al., 2007). The correction method includes filtering a known energy spectrum to account for the attenuation of the derivatives in the PIV and RSM data. The ratio of the measured derivative, denoted by the superscript (m), to the corrected derivative for the longitudinal measurement is given by (Lavoie et al. 2007) as:

9 $$\frac{{\varepsilon }^{m}}{\varepsilon }=\frac{{\langle {\left(\partial {V}_{xx}/\partial {x}_{x}\right)}^{2}\rangle }^{m}}{\langle {\left(\partial {V}_{xx}/\partial {x}_{x}\right)}^{2}\rangle }=\frac{{\iiint }_{-\infty }^{\infty }{B}^{2}\frac{{\mathit{sin}}^{2}\left(\Delta {x}_{x}{k}_{x}/2\right)}{{\left({\Delta x}_{x}/2\right)}^{2}}{\Phi }_{xx}\left(\underline{k}\right){{\rm d}k}_{x}{{\rm d}k}_{y}{{\rm d}k}_{z}}{{\iiint }_{-\infty }^{\infty }{k}_{x}^{2}{\Phi }_{xx}\left(\underline{k}\right){{\rm d}k}_{x}{{\rm d}k}_{y}{{\rm d}k}_{z}}$$

where $\varepsilon$ is the turbulent energy dissipation rate, defined by (Hinze, 1975) as:

10 $$\varepsilon =15\nu \langle {\left(\frac{\partial {V}_{x}}{\partial x}\right)}^{2}\rangle$$

and $B$ is the spatial spectral filtering function given by:

$$B=\frac{8}{\left({wk}_{x}\right)\left(h{k}_{y}\right)\left(s{k}_{z}\right)}$$

11 $${\rm sin}\left(\frac{{k}_{x}w}{2}\right){\rm sin}\left(\frac{{k}_{y}h}{2}\right){\rm sin}\left(\frac{{k}_{z}s}{2}\right)$$

and

12 $${\Phi }_{{\rm xx}}\left(\underline{k}\right)=\frac{E(k)}{{4\pi k}^{-4}}\left({k}^{2}{\delta }_{{\rm xx}}-{k}_{x}{k}_{x}\right)$$

where $\underline{k}$ is the wave number vector with a magnitude $k$, ${\delta }_{xx}$ is the Kronecker delta and ${k}_{x},{k}_{y}$ and ${k}_{z}$ are the wave vector components in the $x,y$ and $z$ directions. The variable $w, h, s,\Delta {x}_{x}$ corresponds to the width, height and depth of the PIV interrogation volume and the separation between PIV vectors, respectively. $E(k)$ is the 3D energy spectrum, which is defined by (Lin, 1972) as:

13 $$E\left( k \right) = \alpha u_{\eta }^{2} \eta \left( {\left( {k\eta } \right)^{{ - 5/3}} + \left( {k\eta } \right)^{1} } \right) \times \exp \left[ { - \alpha \left( {\frac{3}{2}\left( {k\eta } \right)^{{4/3}} + \left( {k\eta } \right)^{2} } \right)} \right]$$

With $\alpha$= 1.8,${u}_{\eta }$ is the Kolmogorov velocity scale, $={\left(\nu \varepsilon \right)}^{1/4}$. According to (Pasquier et al., 2007; McComb, 1990), the Taylor length scale, $\lambda$, is related to the turbulence dissipation rate, $\varepsilon$, by:

14 $$\lambda ={\left(15\nu \langle {{V}_{x}}^{2}\rangle /\langle \varepsilon \rangle \right)}^{2}$$

where $\nu$ is the kinematic viscosity, and values of which were obtained from (Morley, 2005) and $\langle \rangle$ denote time averaging. The corresponding Reynolds number is given by,${ R}_{\lambda } =\lambda {u}^{\prime}/\nu$. The Kolmogorov length scale $\eta$ is given by (McComb, 1990):

15 $$\eta ={\left({\nu }^{3}/\varepsilon \right)}^{1/4}$$

The corrected dissipation rate $\varepsilon ,$ was first evaluated using equations (10) to (13) and then substituted into Eqs. (14) and (15), respectively, to calculate Taylor and Kolmogorov length scales. Using such method, values of the different length scales and corresponding Reynolds numbers are obtained and tabulated in Table 2 for the fan speed range 1000–6000 rpm, for dry atmospheric air temperatures and pressures.

**Table 2:** Values of the length scales and corresponding Reynold numbers, for all fan speeds at atmospheric temperature and pressure, using PIV measurements

Fan speed (rpm)	${R}_{{\rm L}}$	${R}_{\lambda }$	$\lambda$(mm)	$\eta \times 5$(mm)
1000	1615	220.2	2.01	0.88
2000	3360	317.4	1.08	0.51
3000	4943	385.0	0.73	0.38
4000	6300	434.9	0.53	0.30
5000	7956	488.7	0.44	0.26
6000	10274	555.4	0.38	0.23

Figure 10 shows $\lambda$ and *η* decrease with increasing fan speed. They exhibit similar decreases with the fan speed. Since the largest scale $L$ is fixed by the geometric dimensions of the vessel, increasing the Reynolds number (i.e., increasing the fan speed) causes the generation of ever finer local dissipation scales. Again, good consistence has been observed between the results of PIV and RSM and emphasizes the negligible effect of the third dimension velocity on the PIV measurements and the ability of the PIV to reproduce the experimental measurements.

The energy spectra, $E$, were also investigated using both PIV measurements and RSM modeling. The temporal energy spectra of the velocity fluctuations u and v were computed, using a procedure similar to that outlined in (Doron et al. 2001), for each interrogation window and then averaged over all interrogation windows within the velocity map.

**Fig. 11:**
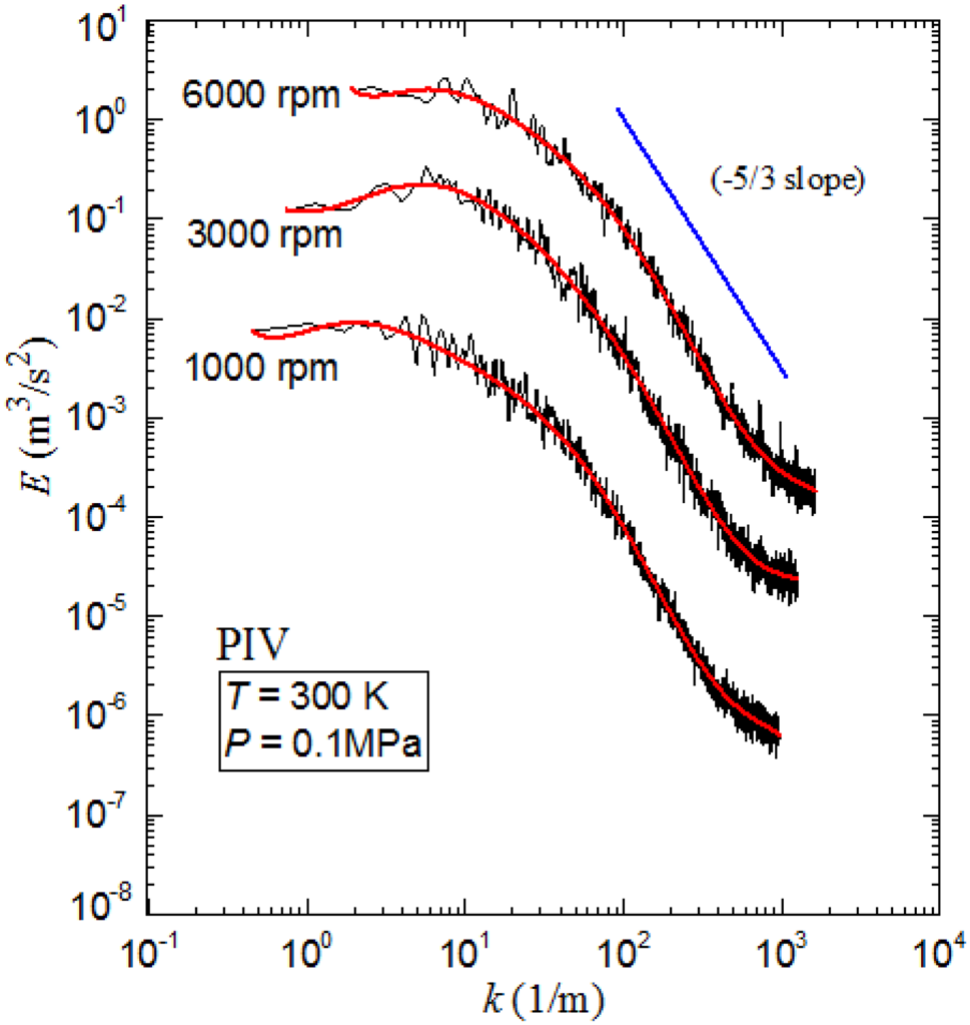
PIV measured turbulent kinetic energy spectra at fan speeds 1000, 3000 and 6000 rpm with a pressure 0.1 MPa and temperature 300 K

Figure 11 shows average values of energy spectra, $E$, of the two components ${E}_{V{\rm xx}}$ and ${E}_{V{\rm yy}}$, for three fan speeds of 1000, 3000 and 6000 rpm. This figure portrays an evident linear relation between energy spectral density and wave number, k. Furthermore, comparison with the -5/3 slope’s reference line implies good agreement to Kolmogorov’s 5/3 law (Fragner, et al. 2015), which attests, once again, to the isotropy assumption.

**Fig. 12:**
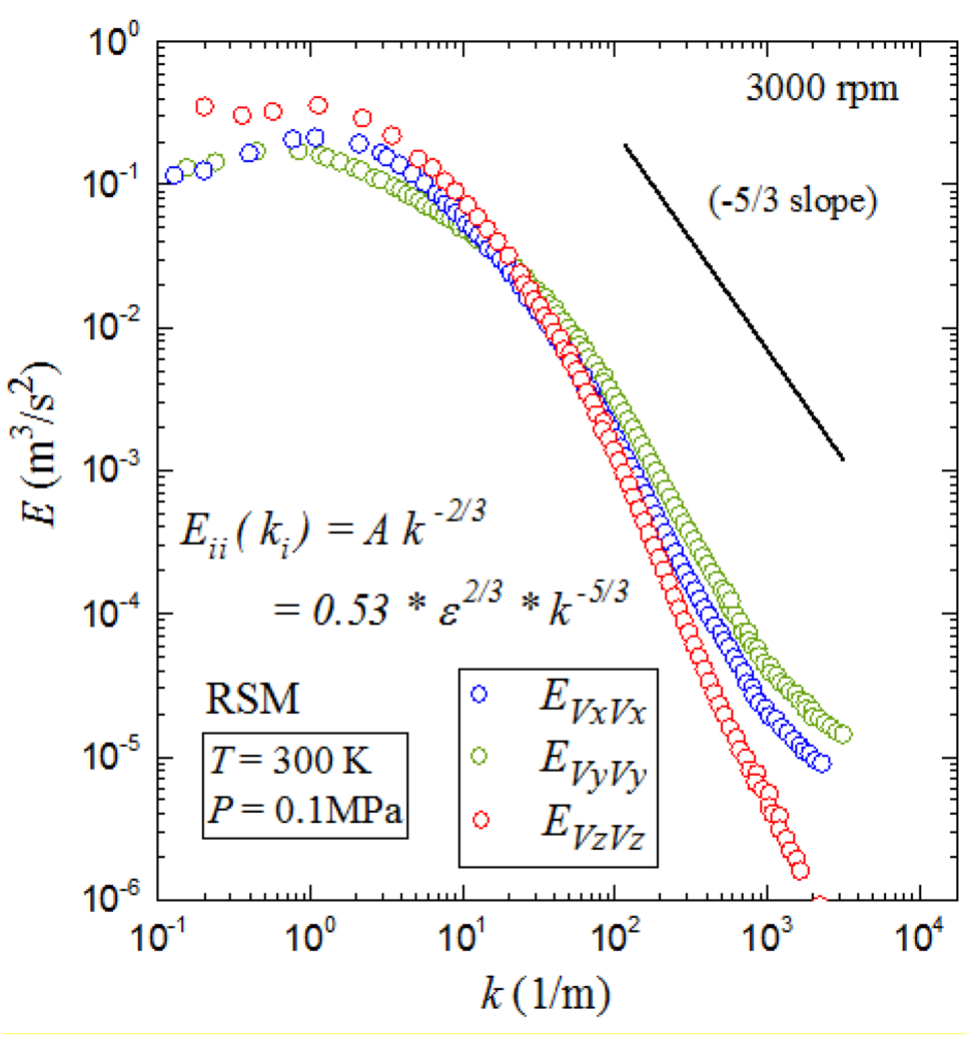
Predicted turbulent kinetic energy spectra at fan speed 3000 rpm, pressure 0.1 MPa and temperature 300 K

Figure 12 shows the computed energy spectra from RSM , $E$, of the three components ${E}_{V{\rm xx}}$,${E}_{V{\rm yy}}$, and ${E}_{V{\rm zz}}$ for a fan speed of 3000 rpm. This figure shows that the three components are consistent. Comparing the energy spectra determined from the PIV measurements, Fig. 11, with the computed energy spectra from RSM, in Fig. 12, shows excellent agreement between both results. This confirms the ability of the RSM model to reproduce the experimental results.

### Influence of temperature and pressure on the turbulent statistics

The previous sections provided characterization of the turbulent flow field in the vessel at atmospheric conditions. Since the vessel was designed to study turbulent combustion at high initial temperature and high initial pressure, close to those encountered in internal combustion engines, an accurate characterization of the turbulent flow at such conditions is required to enable better analysis of the flame/turbulence interactions.

**Fig. 13:**
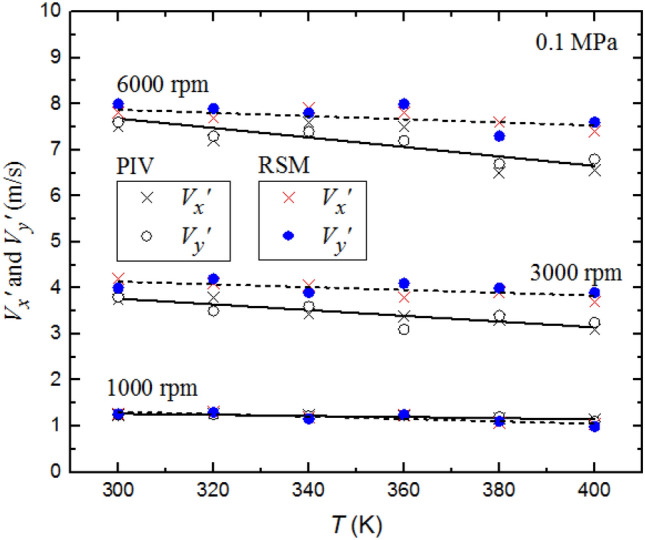
Comparison of predicted and measured the rms turbulent velocities ${V}_{x}^{{\prime}}$ and ${V}_{y}^{{\prime}}$ as a function of temperature at different fan speeds with a pressure 0.1 MPa. The discrete data points are accompanied by the best fit curves. Solid curves for experimental data, broken curves for predicted data

Figure 13 shows the effects of temperature on the temporal rms velocities $V_{x} ^{\prime }$ and $V_{y} ^{\prime }$ at fan speeds of 1000, 3000 and 6000 rpm at 0.1 MPa. Both PIV and RSM results show that the values of both $V_{x} ^{\prime }$ and $V_{y} ^{\prime }$ are close, at the different fan speeds, regardless of increasing temperature. This suggests the maintenance of a homogeneous isotropic structure of the flow with increasing temperature. In contrary to the small effect of temperature on the PIV values of $V_{x} ^{\prime }$ and $V_{y} ^{\prime }$ at 1000 and 3000 rpm, the increase in temperature decreases values of $V_{x} ^{\prime }$ and $V_{y} ^{\prime }$ by up to 12% of the value at 300 K, at 6000 rpm. The RSM results show less effect of the temperature on $V_{x} ^{\prime }$ and $V_{y} ^{\prime }$ by up to 5% of the value at 300 K, at 6000 rpm. Since it has been observed that $L$ remains independent of the temperature and pressure, this might be associated with the variation of $\nu /\lambda$.

**Fig. 14:**
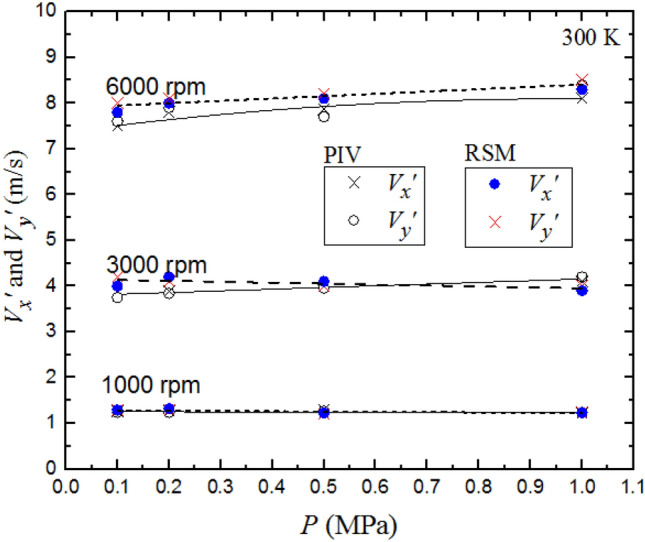
Comparison of predicted and measured the rms turbulent velocities ${V}_{x}^{{\prime}}$ and ${V}_{y}^{{\prime}}$ as a function of pressure at different fan speeds with a temperature 300 K. The discrete data points are accompanied by the best fit curves. Solid curves for experimental data, broken curves for predicted data

Figure 14 shows the effect of pressure on the temporal rms velocities $V_{x} ^{\prime }$ and $V_{y} ^{\prime }$ at fan speeds 1000, 3000 and 6000 rpm at 300 K. Very small increases are observed in the PIV values $V_{x} ^{\prime }$ and $V_{y} ^{\prime }$ with increasing *P*, while the pressure effect was negligible on the computed values of $V_{x} ^{\prime }$ and $V_{y} ^{\prime }$ with increasing *P.* The largest effect was at 6000 rpm. The increase in $V_{x} ^{\prime }$ and $V_{y} ^{\prime }$ , at the highest velocity, might be associated with the small decrease in kinematic viscosity with increasing *P*.

**Fig. 15:**
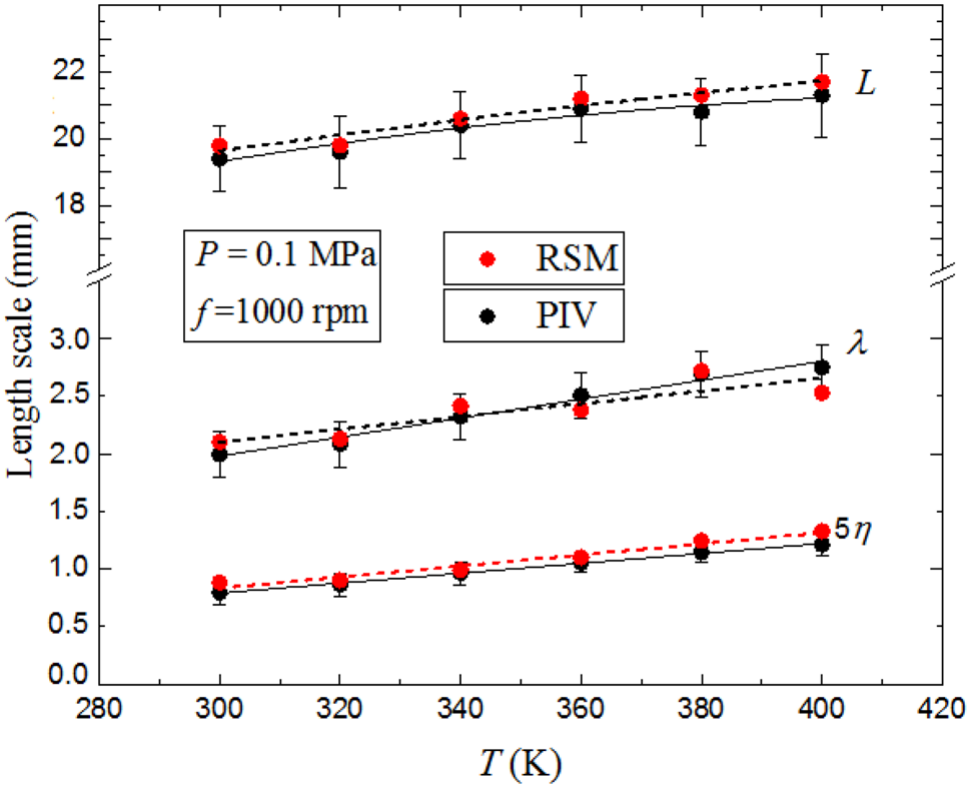
Comparison of predicted and measured the integral length scale, *L,* Taylor length scale, λ, and five times Kolmogorov length scale, 5η, as a function of temperature at fan speed 1000 rpm and pressure 0.1 MPa. The discrete data points are accompanied by the best fit curves. Solid curves for experimental data, broken curves for predicted data

Figure 15 highlights the effect of initial pressure the working fluid on $L$, $\lambda$ and $\eta$ at 300  K, for a fan speed of 1000 rpm. Noticeably, $L$ slightly increases with increasing temperature, in the range ± 10 % from the mean value previously calculated at different fan speeds, in Fig. 10. The PIV results are consistent with the computed values. It can be reasonably concluded that because $L$ is associated with the largest turbulence scales, it can be varied only by the variation of the geometric aspect of the experimental configuration or possibly by changing the profile blades geometries, such as changing the blade pitch angle of the impeller. The Taylor length scale, λ, and Kolmogorov length scale, $\eta$, are increased by up to 47 and 36.5%, respectively, of their values at 300 K with up to 5% differences from the computed values. The fluctuating rate of strain is related to ${u}^{\prime}/\lambda$, in which $\lambda$ is the Taylor scale. This is also related to the turbulence dissipation rate, $\varepsilon$, by $\varepsilon =\nu {{u}^{{\prime}}}^{2}/{\lambda }^{2}$ (McComb, 1990). A key expression relating $\lambda$ to $L$ is (McComb, 1990):

16 $$\uplambda /{\rm L}={\left(15/A\right)}^{1/2}{{\rm R}}_{{\rm L}}^{-1/2}=\left(15/{\rm A}\right){{\rm R}}_{\uplambda }^{-1}.$$

Here $A$ is a numerical constant of order unity,${R}_{L}=L{u}^{\prime}/\nu$ [40] and ${ R}_{\lambda }=\lambda {u}^{\prime}/\nu$[30], where $\nu$ is the kinematic viscosity, values of which were obtained from (Morley, 2005). Equation (16) was confirmed by (Goulier et al. 2017a, b), in which two-dimensional PIV to measure turbulence in a near-spherical vessel, with values of ${R}_{L}$ up to 711. They found equation (16) to hold, with an experimental value of $A$ = 0.98. The Taylor scale correlated with the electrical power input to the turbulence-producing fans. With $\varepsilon =A{u^{\prime}}^{3}/L$ (McComb, 1990), in which *A* is a numerical constant, of order unity. The Kolmogorov scale, $\eta = {\left({\nu }^{3}/\varepsilon \right)}^{1/4}$, is then related by:

17 $$\uplambda /\upeta = {15}^{1/2}{({R}_{L}/{\rm A})}^{1/4}.$$

As shown in Fig. 15, $\lambda$ and *η* increases with increasing temperature. Since $L$ is nearly independent of temperature, such increase in both λ and $\eta$ is related to the increase in the kinematic viscosity, dominated by molecular collisions as the temperature increases, which in turn yields a decrease in ${R}_{L}$, in Eqs. (16) and (17).

**Fig. 16:**
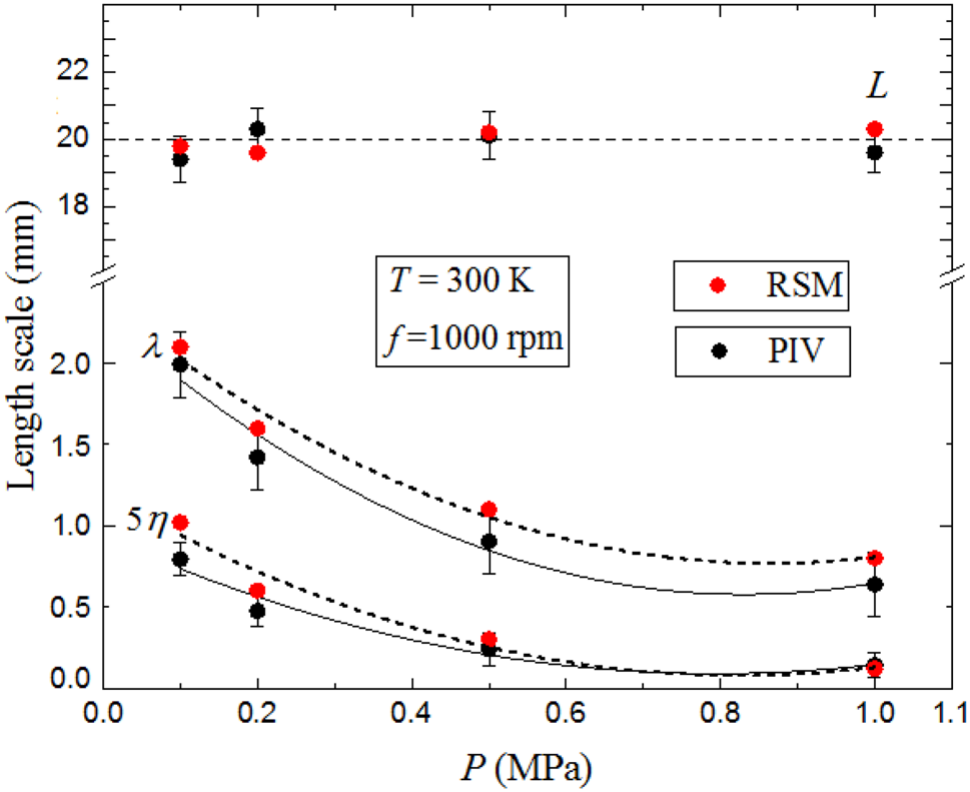
Comparison of predicted and measured the integral length scale, *L,* Taylor length scale, λ, and five times Kolmogorov length scale, 5η, as a function of pressure at fan speed 1000 rpm and temperature 300 K. The discrete data points are accompanied by the best fit curves. Solid curves for experimental data, broken curves for predicted data

As shown in Fig. 16, the variation of $L$, $\lambda$ and $\eta$ with initial pressure, P, is at 300 K for a fan speed of 1000 rpm. This shows the integral length scale, $L$, to be independent of pressure, with a good agreement between PIV and RSM. Both values of $\lambda$ and $\eta$ decrease, with increasing the pressure. The variation of λ follows the power law ${P}^{-1/2}$, and the Kolmogorov length scale, $\eta$, varies as ${P}^{-3/4}$ , implying that increasing the pressure generates smaller eddies and consequently smaller length scales. For all pressures, the computed values of $\lambda$ and $\eta$ are higher than those determined from the PIV measurements, within the range of 4–10%.

### Turbulent flame propagation

The reported facility is specially designed to do several investigations in turbulent combustion and to avoid several problems, associated with the smaller vessels. These problems, like wall confinement and radiation exchange, cause high uncertainty in the measurements of the combustion characteristics. The proposed turbulence statistics prove that the present vessel is suitable for generating a nearly homogeneous and isotropic turbulent flow fields that can be justifiably utilized in future studies.

This vessel has also many features that make it suitable for measuring turbulent burning velocities of both gaseous and vaporized liquid fuels at elevated initial pressures and temperatures with a wide range of turbulence intensities. The flow within the central volume of this vessel is close to homogeneous, isotropic, turbulence with no mean flow, as shown proposed. The equivalence ratio, pressure and temperature can be controlled over a wide range of conditions. Also, only a small amount of fuel is required and the combustion is less affected by complex feedback mechanisms in more complex systems, such as internal combustion (IC) engines. The operating range is also not limited by flashback or blow off, and the flame is more stable compared to those of burners. Furthermore, it allows for various imaging techniques to be used, such as high-speed digital schlieren, and more complex flow field imagining techniques, such as particle image velocimetry (PIV). To demonstrate the capabilities of the vessel, brief results of a few turbulent premixed combustion experiments are reported here after. Such techniques allow us to improve our understanding of combustion and to gain a better understanding regarding flame/flow interactions subjected to engine-like conditions.

**Fig. 17:**
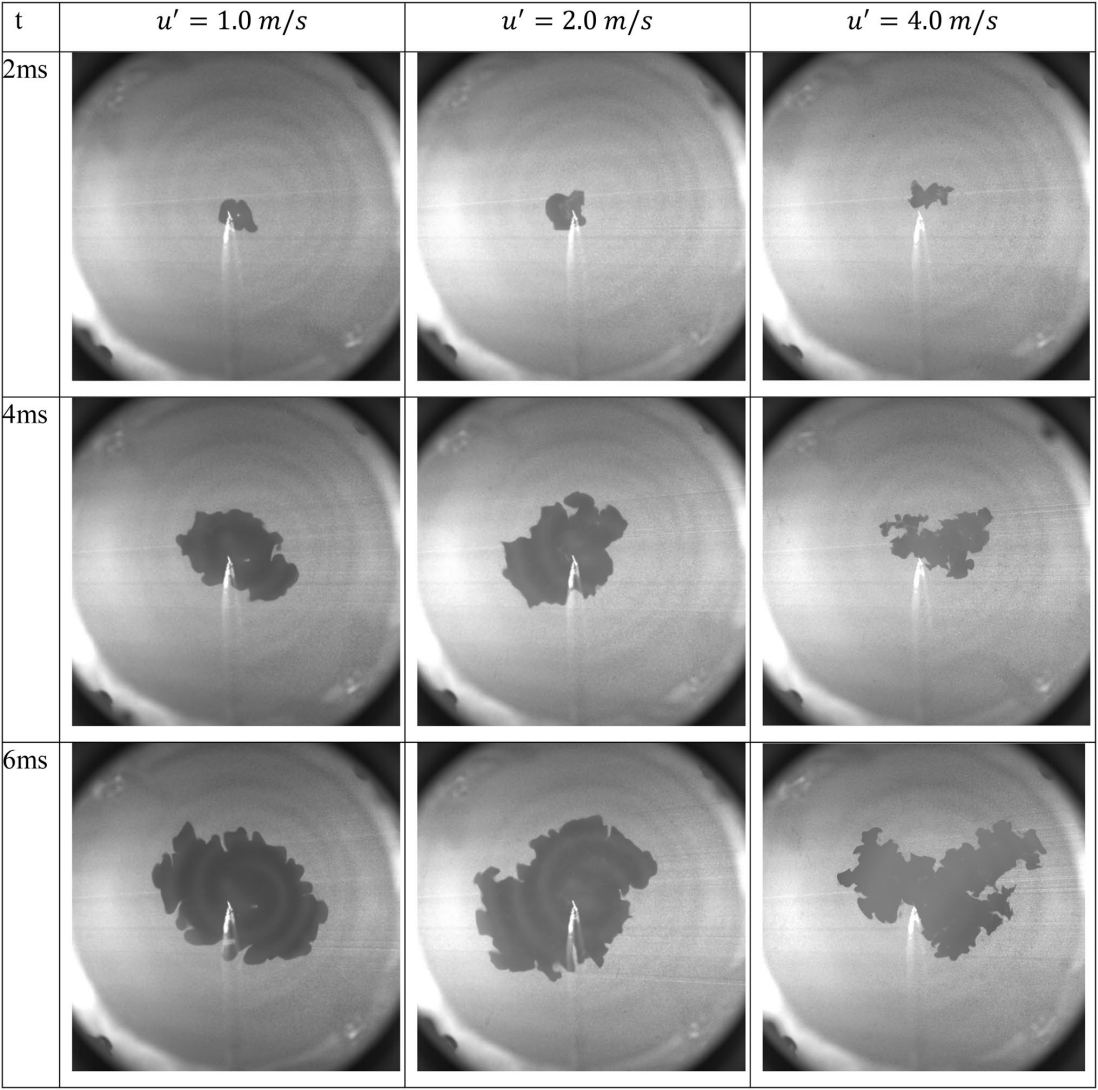
Instantaneous PIV raw images of CH_4_/air premixed flame propagation, at different rms turbulent velocities, $u^{\prime}$ = 1, 2 and 4 m/s, *φ* =1.0 at 0.1 MPa and 300 K

**Fig. 18:**
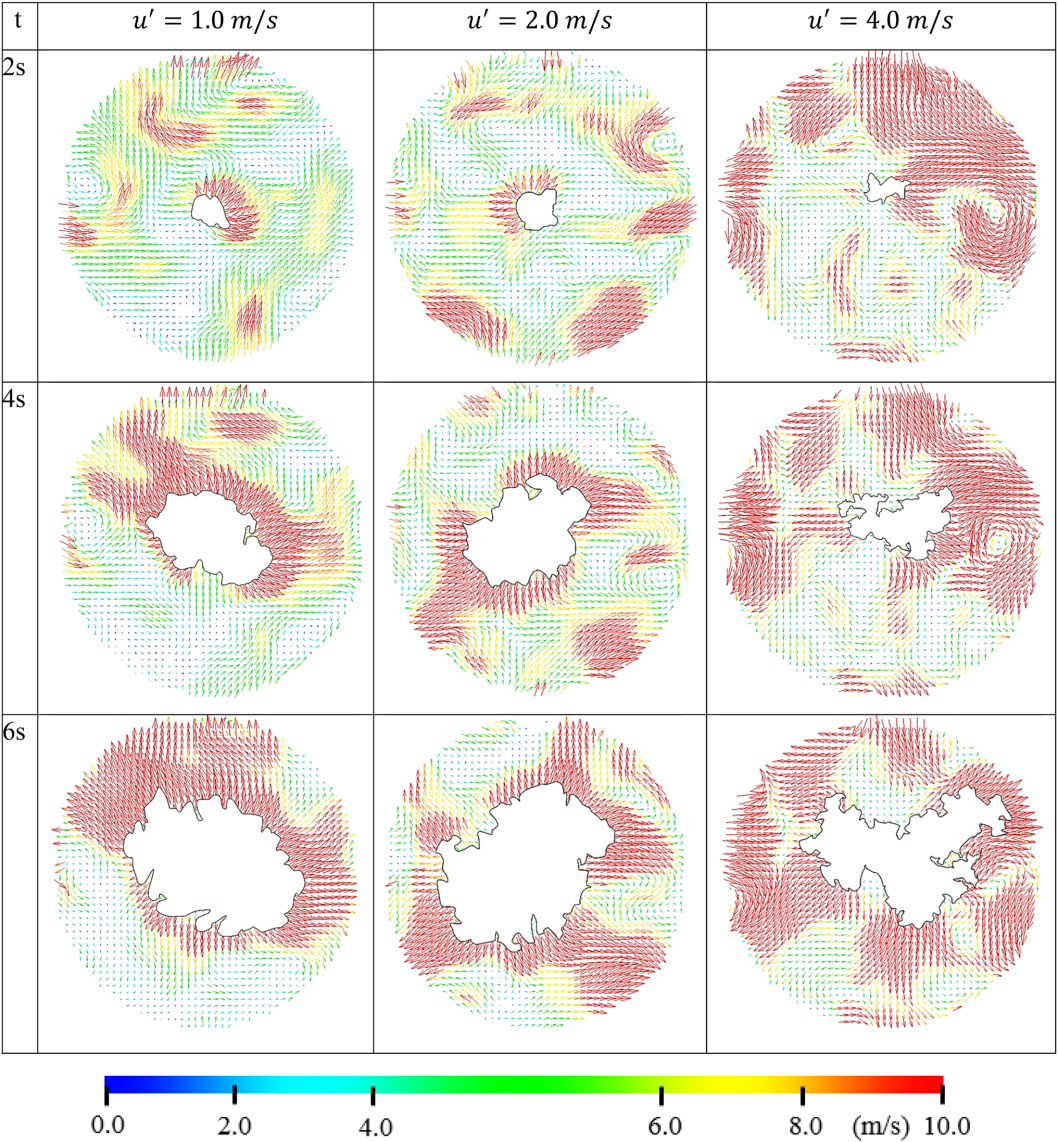
Instantaneous velocity vectors, corresponding to the raw image in Fig. 17

Figures 17 and 18 show a selection of Mie scattering raw images and the corresponding vectors maps, respectively, of stoichiometric methane/air flames at an initial pressure of 0.1 MPa and initial temperature of 300 K for $u^{\prime }$ = 1, 2 and 4 m/s, respectively. The time, $t$, is the elapsed time after ignition. One representative experiment of five is shown here, at each $u^{\prime }$.

It is clear that the interaction between the unburned flow field and the flame front changes significantly with $u^{\prime }$. As $u^{\prime }$ increases, the propagation of the flame changes from something that is loosely spherical in nature (at $u^{\prime }$= 1 m/s) to something highly deformed and convoluted (at $u^{\prime }$ = 4 m/s). It is also clear that the existing flow structures before ignition are different at each $u^{\prime }$ and the flame front responds to these structures from an early stage of the flame propagation. Some sections of the flow are initially moving away from the point of ignition. These sections drag the flame front along with the flow, making it appear that the flame propagates aggressively in that direction.

This phenomenon is somewhat clear in Fig. 17 at $u^{\prime }$= 4 m/s. In contrast, where the flow is moving toward the flame, the distance that the flame travels noticeably arrested. Clearly, the response of the flame to the flow movement is due to the relative difference between the burning velocity of the mixture and the flow velocity ahead of the flame. The laminar burning velocity, ${u}_{l}$, for an outwardly propagating flame through stoichiometric methane/air, at an initial pressure of 0.1 MPa, is 0.358 m/s (Bradley et al. 2019).

If this burning velocity is compared with the flow velocity at each $u^{\prime }$, it is clear that the local change in flow velocity is significantly higher than the burning velocity. As a result of this difference, the flow is able to wrinkle and displace the flame front before a particular flow structure is consumed by the flame. For the case of $u^{\prime }$= 1.0 m/s, the flow velocity is not substantial enough to seriously distort the flame front and convolute it significantly from its spherical nature and the flame front is only slightly wrinkled. As $u^{\prime }$ increases, the flow velocity fluctuations ahead of the flame front are also increase and become significantly higher. As a result, the flame is distorted and moved by the flow, wrinkling and stretching the flame front beyond recognition from its spherical natures. It is important to note that the relevant Lewis numbers are all close to unity. This diminishes any chances of diffusional thermal instability.

For all $u^{\prime }$, as the flame propagates, the velocity of unburned gas ahead of the flame is being pushed by outward by the expanding burned gases. This phenomenon is most noticeable at $u^{\prime }$= 1.0 m/s, due to the relatively slow moving structures, but it is still evident even for $u^{\prime }$= 4.0 m/s. This indicates that there is a symbiotic relationship between the flow structures contained within the reactants and the propagating flame front. The flame increases the velocity ahead of its front. This alters the flow structure which in turn alters the flame structure, in agreement with the seminal work of (Bradley et. al. 2021). They have shown that rapid gaseous expansion, generated by the combined influences of burning rate and volumetric expansion during spherical explosion, generates an additional radial outward velocity, from the wrinkled flame surface, and additional turbulent kinetic energy.

## Conclusions

The flow fields in a large fan-stirred vessel have been investigated both numerically and experimentally using 3D CFD and PIV techniques. The predicted and measured turbulence statistics at the central plane of the vessel have been compared and discussed under different conditions. An acceptable agreement between the PIV and RSM results is obtained. The RSM simulation has shown its ability to reproduce similar behaviors for the turbulence structure and intensity as observed in experiments for different fan speed between 1000 and 6000 rpm. Both RSM and PIV results have also shown that the mean velocity of turbulent flow is relatively small compared with the rms turbulent velocity .

The results also revealed that the vessel can generate a region of homogeneity and isotropy in the central area, which decreases with increasing fan speed. The turbulence length and timescales in this area were evaluated under different pressures, temperatures and fan speeds. The integral length scale, $L$, is independent of the pressure and increases slightly with temperature. When $L$ is calculated in the three dimensions using RSM results, same value is obtained. Both the Taylor and Kolmogorov length scales decrease with increasing pressure, $P$, and vice versa with increasing temperature, $T$, due to the variation in the kinematic viscosity induced by the changes in thermodynamic conditions (i.e., $T$ and $P$). Both PIV and RSM results show small effect of temperature and pressure change upon rms velocities, at fan speeds less than 6000 rpm. At higher speeds, these values decrease with increasing temperature, with slight increase in velocities with increasing pressures.

In order to demonstrate the practical importance of the apparatus, preliminary PIV results from turbulent combustion experiments were presented. The results have shown that the radial flame propagation changes the original cold flow turbulence and there is a symbiotic relationship between the flow structures contained within the reactants and the propagating flame front. The flame is affecting the structure of the flow, and the flow is affecting the structure of the flame.

## Data Availability

The data that support the findings of this study are available from the corresponding author upon reasonable request.
